# New Insights into the Biosynthesis Pathway of Polyketide Alkaloid Argimycins P in *Streptomyces argillaceus*

**DOI:** 10.3389/fmicb.2018.00252

**Published:** 2018-02-16

**Authors:** Suhui Ye, Alfredo F. Braña, Javier González-Sabín, Francisco Morís, Carlos Olano, José A. Salas, Carmen Méndez

**Affiliations:** ^1^Departamento de Biología Funcional e Instituto Universitario de Oncología del Principado de Asturias, Universidad de Oviedo, Oviedo, Spain; ^2^Instituto de Investigación Sanitaria del Principado de Asturias, Oviedo, Spain; ^3^EntreChem, S.L., Oviedo, Spain

**Keywords:** type I polyketide synthase, pyridine, piperidine, piperideine, imine reductase, specialized metabolites, regulation, alkaloid

## Abstract

Argimycins P are a recently identified family of polyketide alkaloids encoded by the cryptic gene cluster *arp* of *Streptomyces argillaceus*. These compounds contain either a piperideine ring, or a piperidine ring which may be fused to a five membered ring, and a polyene side chain, which is bound in some cases to an *N*-acetylcysteine moiety. The *arp* cluster consists of 11 genes coding for structural proteins, two for regulatory proteins and one for a hypothetical protein. Herein, we have characterized the post-piperideine ring biosynthesis steps of argimycins P through the generation of mutants in *arp* genes, the identification and characterization of compounds accumulated by those mutants, and cross-feeding experiments between mutants. Based in these results, a biosynthesis pathway is proposed assigning roles to every *arp* gene product. The regulation of the *arp* cluster is also addressed by inactivating/overexpressing the positive SARP-like *arpRI* and the negative TetR-like *arpRII* transcriptional regulators and determining the effect on argimycins P production, and through gene expression analyses (reverse transcription PCR and quantitative real-time PCR) of *arp* genes in regulatory mutants in comparison to the wild type strain. These findings will contribute to deepen the knowledge on the biosynthesis of piperidine-containing polyketides and provide tools that can be used to generate new analogs by genetic engineering and/or biocatalysis.

## Introduction

Alkaloids are a group of natural products synthesized by plants, animals, and microorganisms that show a broad structural diversity and the presence of a basic nitrogen atom as a common feature (Cushnie et al., 2014; Sigrist et al., 2015). This family of compounds shows significant bioactivity, in some cases beneficial (antimalarial, antitumor, antiasthma, antihypertensive, anti-arrhythmic, analgesic), but in others harmful for humans and animals (Cushnie et al., 2014; Sigrist et al., 2015). In addition, they seem to play different roles in producer organisms. In microorganisms, alkaloids have been involved in growth and colony development, self-defense or as autoinducers (Heeb et al., 2011; Cushnie et al., 2014; Ye et al., 2017). Attending to the chemical structure there are two classes of alkaloids: heterocyclic or typical alkaloids and non-heterocyclic/atypical/protoalkaloids bearing nitrogen atoms in a side chain (Cushnie et al., 2014). Some of the most important alkaloids contain a piperidine ring, and in the so called true alkaloids these rings derive from amino acids. However, some piperidine-containing alkaloids or piperidine pseudoalkaloids from polyketide origin acquire their nitrogen atoms via transamination reactions (Sigrist et al., 2015).

Actinomycetes are a group of bacteria that have attracted the attention of academic and industrial researchers, because they produce a plethora of natural products displaying antibiotic, antitumor, immunosuppressant or anthelmintic activities (Bérdy, 2012; Newman and Cragg, 2016). Most of these compounds are peptides (ribosomal and non-ribosomal synthesized), polyketides or terpenes. Particularly, several piperidine-containing alkaloids of polyketide origin have been isolated from actinomycetes, such as coelimycin P (Gómez-Escribano et al., 2012), cyclizidine (Freer et al., 1982), iromycins (Surup et al., 2007), latumcidin (also known as abikoviromycin; Seto et al., 1973), piericidins (Zhou and Fenical, 2016), strepchazolins (Yang et al., 2017), streptazolins (Mayer and Thiericke, 1993; Puder et al., 2001), streptazones A–D (Puder et al., 2000), streptazone E (Liu et al., 2013), or streptopyridines (Groenhagen et al., 2014). These compounds show a range of activities such as antimicrobial, antiviral, cytotoxic, decreasing blood pressure and cholesterol biosynthesis, or analgesic (Umezawa et al., 1951; Terashima et al., 1970; Grabley et al., 1991; Puder et al., 2001). However, information about their biosynthesis pathways is quite scarce. Thus, only the biosynthesis gene clusters for coelimycin P, cyclizidine and streptazone E have been identified so far (Gómez-Escribano et al., 2012; Huang et al., 2015; Ohno et al., 2015). Biosynthesis of these compounds involves a Type I or modular polyketide synthase (PKS). These are macroenzyme complexes typically organized into modules, which are responsible for a single elongation cycle and contains a β-ketoacyl synthase (KS), an acyltransferase (AT) and an acyl carrier protein (ACP) domain. In addition, these modules can contain optional domains such as ketoreductase (KR), dehydratase (DH) or enoyl reductase (ER) that modify the β-keto group after an elongation event (Weissman, 2015).

**FIGURE 1:**
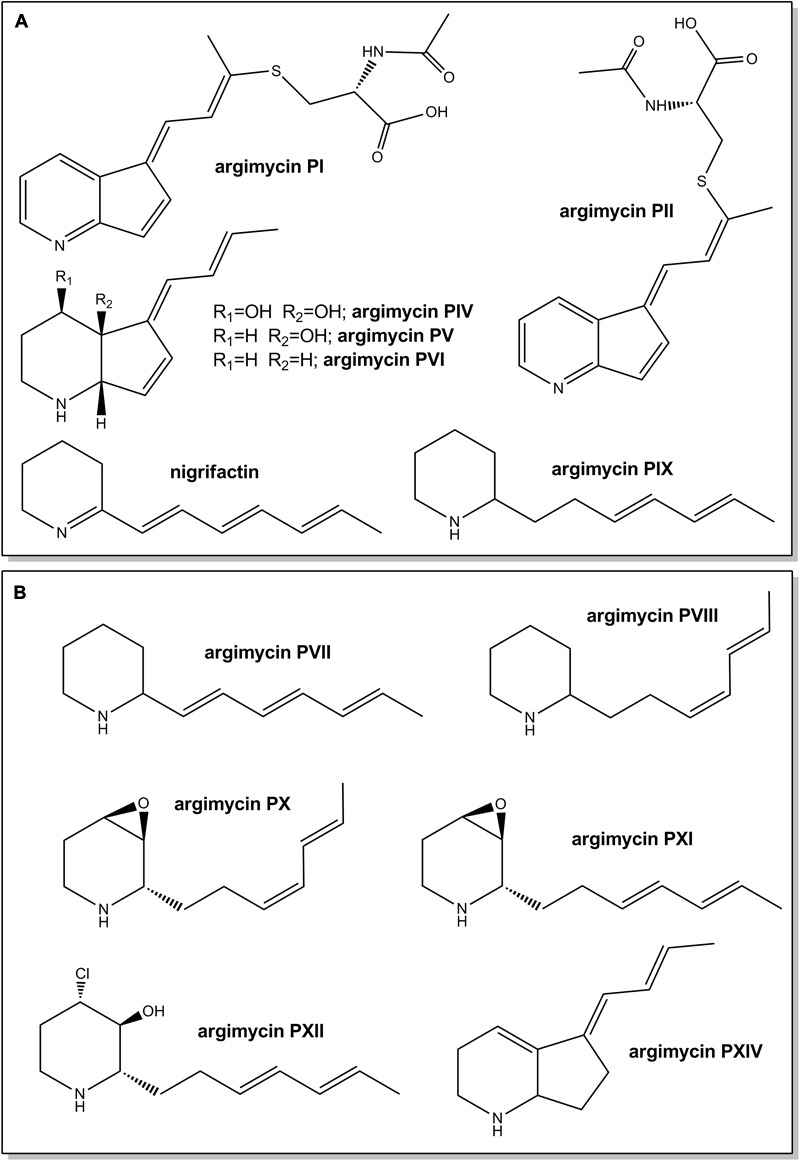
Chemical structures of argimycins P previously identified in *Streptomyces argillaceus* wild type strain **(A)** and new ones identified in the wild type strain and/or in *arp* mutants (this work) **(B)**. Relative configuration is shown for argimycins PI, PII, PIV, PV, PVI, PX, PXI, and PXII.

Argimycins P (**Figure 1A**) comprise a group of polyketide alkaloid compounds produced by *Streptomyces argillaceus* ATCC 12956 that show either a piperideine ring, or a piperidine ring fused or not with a five-membered ring, and a polyene side chain. In addition, some argimycins P contain an *N*-acetylcysteine moiety. Very recently, we have reported the isolation and chemical characterization of these compounds that seem to play some role in both growth and colony development in the producer strain (Ye et al., 2017). In addition, we also reported the identification of the argimycins P biosynthesis gene cluster *arp* and showed that biosynthesis of nigrifactin, a putative intermediate containing a piperideine ring, requires the ArpP PKS and the ArpN aminotransferase (Ye et al., 2017). Herein we describe new insights into the argimycin P biosynthesis pathway through the inactivation of *arp* genes putatively involved in post-piperideine ring formation biosynthesis steps, the identification and characterization of the compounds produced by each mutant, and cross-feeding experiments between mutants. In addition, we also address the regulation of argimycins P biosynthesis by studying the expression pattern of the *arp* genes in mutants in specific regulatory genes in comparison with the wild type strain.

## Materials and Methods

### Strains, Culture Conditions, Plasmids and DNA Manipulations

*Streptomyces argillaceus* ATCC 12956 was used as source of DNA to generate mutants and for argimycins P production experiments. The argimycin P-non producers *S. argillaceus* MARPPIII (PKS-minus mutant), *S. argillaceus* MARPN (aminotransferase-minus mutant), *S. argillaceus* DARPO-HII (mutant with a deletion from *arpO* to *arpHII*), *S. argillaceus* MARPRI (positive regulatory *arpRI*-minus mutant) and *S. argillaceus* MARPRII (negative regulatory *arpRII*-minus mutant) (**Table 1**) (Ye et al., 2017) were used in co-synthesis and/or bioconversion experiments, and to quantify argimycins P production. *Escherichia coli* DH10B (Invitrogen) and *E. coli* ET12567/pUB307 (Kieser et al., 2000) were used as cloning hosts for plasmid propagation and for conjugation experiments, respectively. MA and SM10 media (Fernández et al., 1998; Ye et al., 2017) were used for sporulation and argimycins P production by *S. argillaceus*, respectively. When required, antibiotics were added to media at the following final concentrations: ampicillin (100 μg/mL), kanamycin (50 μg/mL), nalidixic acid (25 μg/mL), apramycin (25 μg/mL), and thiostrepton (50 μg/mL). Plasmids pCRBlunt (Invitrogen) and pUO9090 (M. C. Martín, unpublished results) were used for subcloning. Plasmid pHZ1358 (Sun et al., 2002) was used for generating mutants by gene replacement. pIAGO (Aguirrezabalaga et al., 2000), pSETE (I. García, personal communication), pSETec and pSETeTc (Cano-Prieto et al., 2015) were used for complementing mutants and overexpressing *arp* genes. DNA manipulations, intergeneric conjugations and transformations were carried out according to standard procedures for *Streptomyces* (Kieser et al., 2000) and for *E. coli* (Sambrook and Russell, 2001). PCR amplifications were done using Herculase (Stratagene) and 5% dimethyl sulfoxide (DMSO). Purified amplicons were sequenced and compared to others in databases. Sequence analyses were carried out using BLAST (Altschul et al., 1997).

**Table 1:** *Streptomyces argillaceus* strains and plasmids used in this work.

Mutant strain	Inactivated gene(s)	Plasmid	Source
MARPN	*arpN*		Ye et al., 2017
MARPPIII	*arpPIII*		Ye et al., 2017
MARPRI	*arpRI*		Ye et al., 2017
MARPRII	*arpRII*		Ye et al., 2017
DARPO-HII	*arpO, arpDHI, arpDHII, arpN, arpK, arpHI, arpHII*		Ye et al., 2017
MARPDHI	*arpDHI*	pHZMutorf6	This work
MARPDHII	*arpDHII*	pHZMutorf7long	This work
MARPHI	*arpHI*	pHZMutorf9a	This work
MARPHII	*arpHII*	pHZMutorf9b	This work
MARPK	*arpK*	pHZMutorf9	This work
MARPO	*arpO*	pHZMutorf5	This work
MARPX	*arpX*	pHZMutorf14	This work

**Recombinant strain**	**Expressed gene**	**Plasmid**	**Source**

MARPDHI+pIAGOorf6bis	*arpDHI*	pIAGOorf6	This work
MARPDHII+pSETETcorf7	*arpDHII*	pSETETcorf7	This work
MARPHI+pIAGOorf9a	*arpHI*	pIAGOorf9a	This work
MARPHII+pIAGOorf9b	*arpHII*	pIAGOorf9b	This work
MARPK+pIAGOorf9bis	*arpK*	pIAGOorf9	This work
MARPO+pIAGOorf5	*arpO*	pIAGOorf5	This work
WT+pSETWEcRI	*arpRI*	pSETEcRI	This work
WT+pSETERII	*arpRII*	pSETERII	This work
DARPO-HII-pSETETorf7	*arpDHII*	pSETETorf7	This work

### Generation of Mutants

Mutants in specific *arp* genes were generated by replacing most of the corresponding genes by an apramycin resistance cassette that was inserted in the same direction of transcription (**Table 1**). Accordingly, several plasmids were constructed in pHZ1358 by PCR amplifying DNA regions flanked the target gene using oligonucleotides described in Supplementary Table S1, and by inserting an apramycin resistance cassette between those fragments (see Supplementary Material). Amplified DNA fragments were checked by DNA sequencing. The resultant constructs were introduced in *S. argillaceus* and apramycin-resistant, thiostrepton-sensitive transconjugants were selected to identify mutants in which the wild type copy of the target gene was replaced by the mutated one. These mutants were confirmed by PCR amplification of the mutated region using oligonucleotides from Supplementary Table S1, followed by sequencing of the amplified DNA fragments.

### Plasmids for Expressing *arp* Genes

Several plasmids were constructed to complement different *S. argillaceus arp* mutants (**Table 1**). To this aim, single *arp* genes were amplified using the corresponding oligonucleotides from Supplementary Table S2, and subcloned under the control of the erythromycin resistance promoter. *arpHI, arpHII, arpO*, and *arpK* were amplified as SpeI-XbaI DNA fragments and subcloned into the XbaI site of pIAGO; *arpDHII* was also amplified as a SpeI-XbaI DNA fragment but subcloned into the XbaI site of pSETeTc; *arpDHI* was amplified as a NheI-XbaI DNA fragment and subcloned into the XbaI site of pIAGO.

Plasmids pSETEcRI and pSETERII were constructed to independently overexpress regulatory genes *arpRI* and *arpRII*, respectively, in the wild type strain (**Table 1**). To construct pSETEcRI, *arpRI* was amplified using oligonucleotides SARP_ATG_Abisbis/378SARP_B (Supplementary Table S2), subcloned into pCR-Blunt, and the fragment released as an EcoRI fragment (using these sites from the vector) to be subcloned into the same site of pSETec, downstream of the erythromycin resistance promoter. To construct pSETERII, *arpRII* was amplified as a BamHI DNA fragment using oligonucleotides SA1701orf4A/SA1701orf4B (Supplementary Table S2) and subcloned into the BamHI site of pSETE, downstream of the erythromycin resistance promoter.

### UPLC Analysis and Purification of Argimycins P

Argimycins P were extracted with *n*-butanol and analyzed by reversed-phase chromatography as previously reported (Ye et al., 2017). Chromatograms were recorded at 400, 272, and 230 nm. For purification purposes, *S. argillaceus* mutant strains were grown by a two-step culture method as previously described (Fernández et al., 1998), using forty 250-mL Erlenmeyer flasks in the production step. Purification of new argimycins P was carried out as previously described (Ye et al., 2017), using isocratic chromatography conditions optimized for each compound. Structures of new compounds were characterized by LC/MS (liquid chromatography mass spectrometry) and Nuclear Magnetic Resonance (NMR) analyses. A combination of 1D (^1^H and ^13^C), and 2D (^1^H-^1^H COSY, TOCSY, HSQC and HMBC) analyses were carried for structural elucidation by NMR.

### Co-synthesis and Bioconversion Experiments

For co-synthesis analyses, 100 μl of seed cultures from each strain were used to inoculate 3 ml of SM10 medium contained into a well of a 24-square deep-well plate. After 24 h of growth, co-synthesis cultures were prepared by combining cultures from two individual cultures (1.5 ml from each) into a well. Samples were harvested after 0, 24, and 48 h of incubation.

For bioconversion experiments with nigrifactin, cultures of the different mutant strains in SM10 medium were fed with nigrifactin (0.1 mM final concentration). Samples were harvested after 24 h of incubation.

### Transcription Analyses

Total RNA was obtained from *S. argillaceus* wild type and *S. argillaceus* mutants MARPRI and MARPRII as previously described (Flórez et al., 2015). This RNA was used as template for gene expression analysis by reverse transcriptase-PCR (RT-PCR) and real time PCR (qPCR). Qualitative gene expression was studied using the SuperScript^®^III^TM^ One-Step RT-PCR system with Platinum^®^ Taq DNA polymerase (Invitrogen). cDNA synthesis was performed using 50 ng or 100 ng of total RNA as template at 50°C for 30 min, followed by heating at 94°C for 2 min. Sample mixtures included 32.2 U of RNA-guard RNase inhibitor (Amersham Biosciences). Amplification conditions were as follows: 33 cycles of 98°C for 15 s; 62°C for 45 s; 72°C for 1 min; and a final extension step at 72°C for 10 min. Oligonucleotides (Supplementary Table S3) were designed within every *arp* gene to produce cDNAs of approximately 350–550 bp. Primers for *arpRI* and *arpRII* were designed to amplify an internal DNA region located upstream of the inserted apramycin resistance cassette. In the case of *arpRII* the cDNA product was of approximately 150 bp. The identity of these fragments was verified by direct sequencing. Negative controls for each pair of primers were carried out with Platinum Taq DNA polymerase (Invitrogen) in the absence of reverse transcriptase. *hrdB* gene expression levels were used to normalize RNA concentration of the tested strains. The RT-PCR products were resolved by 1.4% agarose gel electrophoresis stained with ethidium bromide and visualized using a Gel Doc analyzer (Bio-Rad).

qPCR was used to quantify gene expression of selected genes in mutants MARPRI and MARPRII, in relation to the wild type strain. A total amount of 0.6 μg of RNA was used to synthesize cDNA using the AffinityScript QPCR cDNA synthesis kit (Bio-Rad). qPCRs were carried out on an Stratagene Mx3000P (Agilent Technologies), with a Brilliant II SYBR^®^ green QPCR master mix (Agilent Technologies). Triplicate PCR reactions were carried out for each sample analyzed. *hrdB* was used as housekeeping gene in each sample in order to standardize the results by eliminating variation in RNA and cDNA quantity and quality. Absence of chromosomal DNA contamination was checked by qPCR. Primers (Supplementary Table S3) were designed using the algorithms provided by Primer Express software 2.0 (Applied Biosystems), and their efficiency was calculated based on the slope of a standard curve. To determine amplification specificity an additional dissociation curve analysis was performed after the last cycle, showing in all cases one single peak. PCR results were given as the increase in the fluorescence signal of the reporter dye detected and visualized by the MxPro Software provided with the version 4.1 (Agilent Technologies). Changes in gene expression are represented with respect to the control sample (wild-type strain).

## Results and Discussion

### Generation and Characterization of *S. argillaceus* Mutants in *arp* Genes

The *arp* gene cluster contains 14 genes: 11 for structural proteins (*arpDHI, arpDHII, arpHI, arpHII, arpK, arpN, arpO, arpPI, arpPII, arpPIII*, and *arpT*), two coding for regulatory proteins (*arpRI* and *arpRII*), and one for a hypothetical protein (*arpX*). We have previously shown that formation of the piperideine ring of argimycins P is achieved by expressing the *arpPI, arpPII*, and *arpPIII* genes that encode a hexamodular PKS and the *arpN* aminotransferase gene (Ye et al., 2017). In order to obtain further insights into argimycin P biosynthesis and to assign functions to other *arp* coding genes, a set of gene-knockout experiments were carried out, followed by metabolic profile analyses. Mutants were obtained by replacing the wild type copy of the gene in the *S. argillaceus* chromosome by an *in vitro* mutated one. Several constructs in the unstable plasmid pHZ1358 were generated, in which most of the target *arp* gene was replaced by an apramycin resistance cassette inserted in the direction of transcription of the targeted gene. The resultant constructs were introduced into *S. argillaceus*, and apramycin-resistant thiostrepton-sensitive transconjugants were selected. One mutant for each inactivated gene was selected for further characterization. The occurrence of a double recombination event in each mutant strain was genetically verified by PCR analyses using proper oligoprimers (Supplementary Table S1). In order to determine that no other gene except the target gene was affected, *in trans* complementation of each mutant was carried out using plasmids expressing the corresponding gene and by confirming the recovery of argimycins P production (**Table 1** and Supplementary Figure S1). The mutants were further characterized for production of argimycins P (or analogs) by Ultra Performance Liquid Chromatography (UPLC) and HPLC-MS analyses, and the new compounds accumulated by the mutants purified and characterized.

#### Mutants in *arpDHI* and *arpDHII* Genes

**FIGURE 2:**
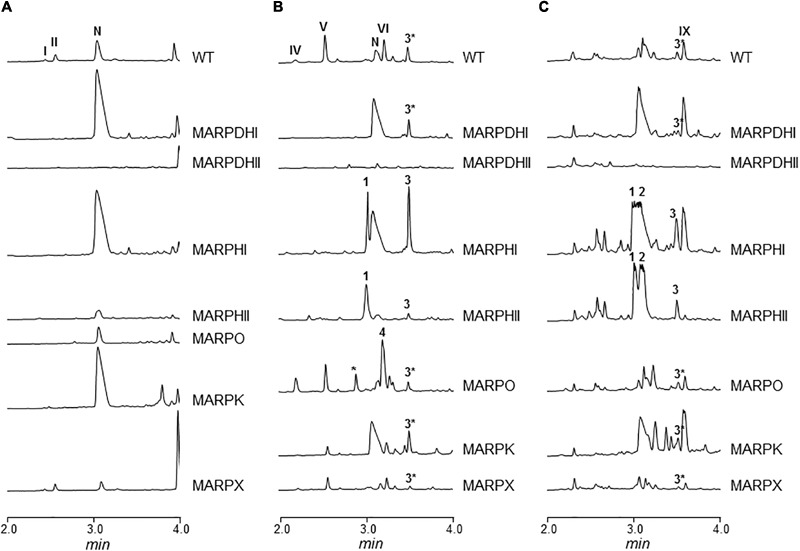
UPLC chromatograms of butanol extracts of *S. argillaceus* wild type (WT) and of *arp* mutant strains. Chromatograms are shown at 400 nm **(A)**, 272 nm **(B)**, and 230 nm **(C)**. Peaks corresponding to the different argimycins P are indicated as follows: argimycins PI and PII (I and II); nigrifactin (N); argimycin PIV (IV); argimycin PV (V); argimycin PVI (VI); argimycin PIX (IX). Peaks 1–4 contain new argimycins P identified in this work: argimycin PX (1); argimycin PXI (2); argimycin PVII and argimycin PVIII (3^∗^); argimycin PVII, argimycin PVIII and argimycin PXII (3); argimycin PXIV (4).

The *arp* gene cluster contains two contiguous genes transcribed in the same direction (*arpDHI* and *arpDHII*) that code for dehydrogenases: ArpDHI is similar to acyl-CoA dehydrogenases, and it has been hypothesized to be involved in the hydroxylation of the piperidine ring and/or in the reduction of the C7–C8 double bond of the polyene chain during argimycins P biosynthesis (Ye et al., 2017). ArpDHII is similar to 6-phosphogluconate dehydrogenases, and it has been proposed to be an imine reductase catalyzing the reduction of the imine group (Ye et al., 2017). Using pHZMutorf6 and pHZMutorf7long, the wild type copy of *arpDHI* and *arpDHII* were replaced by an apramycin resistance cassette, generating mutants MARPDHI and MARPDHII, respectively (**Table 1** and Supplementary Figures S2, S3). Analysis of cultures of mutant MARPDHI (**Figure 2**) revealed production of compounds containing a single piperideine or a piperidine ring (nigrifactin and argimycin PIX; **Figure 1A**) and the disappearance of argimycins P compounds containing the five-membered ring (**Figure 1A**). Further analysis of this mutant showed that it also produced argimycins PVII and PVIII (peak 3^∗^ in **Figure 2**; see below). These results indicated that ArpDHI is involved in an early biosynthesis step of argimycins P prior to the five-membered ring formation. Since argimycin PVIII and PIX are produced by mutant MARPDHI, this ruled out ArpDHI from the reduction of the C7–C8 double bond of the polyene side chain and in the reduction of the imine.

**FIGURE 3:**
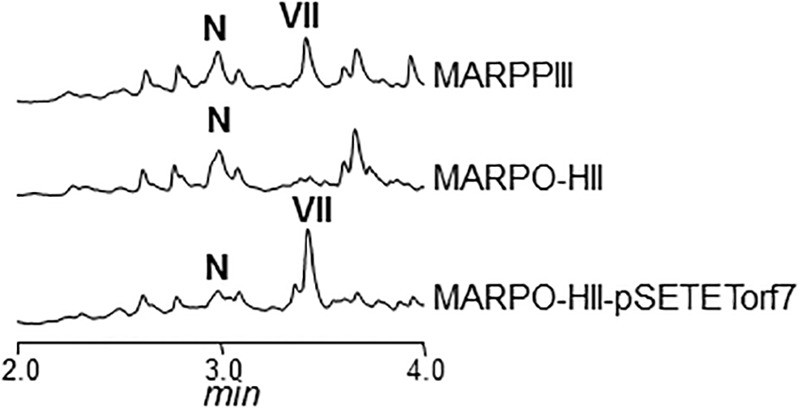
UPLC chromatograms of butanol extracts of bioconversion products of nigrifactin (N) using different *S. argillaceus* mutant strains. Chromatograms are shown at 272 nm. Peak VII corresponds to argimycin PVII.

Cultures of MARPDHII contained traces of unidentified argimycin P-like compounds and a small amount of nigrifactin (**Figure 2B**). This result reinforced the hypothesis of ArpDHII acting in an early biosynthesis step as an imine reductase, most likely before ArpDHI. Based on this result, bioconversions experiments were designed to confirm this activity. Accordingly, nigrifactin which contains an imine group was fed to cultures of the argimycin P non-producer *S. argillaceus* MARPPIII (PKS-minus mutant) to determine if nigrifactin could be converted to other argimycins P. After 24 h of incubation, a new peak was detected (peak VII in **Figure 3**) that showed the same retention time, mass and absorption spectrum as argimycin PVII (**Figure 1B**; see below). This bioconversion indicated that the imine group in nigrifactin is reduced to the amino group in argimycin PVII. Since none of the argimycins P bearing the five-membered ring was detected, this result also suggested that neither nigrifactin nor argimycin PVII were biosynthetic intermediates for the formation of the five-membered ring compounds. To determine if the imine reductase coding gene was an *arp* gene, nigrifactin was fed to *S. argillaceus* DARPO-HII (a mutant with a deletion from *arpO* to *arpHII*; **Table 1**). Argimycin PVII could not be detected (**Figure 3**), indicating that one of the *arp* deleted genes in this mutant was responsible for reduction of the imine. Finally, to identify this *arp* gene, nigrifactin was fed to *S. argillaceus* DARPO-HII expressing *arpDHII* (*S. argillaceus* DARPO-HII-pSETETorf7; **Table 1**). In this case, argimycin PVII was detected (**Figure 3**). These results confirmed the function of ArpDHII as the imine reductase involved in argimycins P biosynthesis.

#### Mutants in *arpHI* and *arpHII* Genes

**FIGURE 4:**
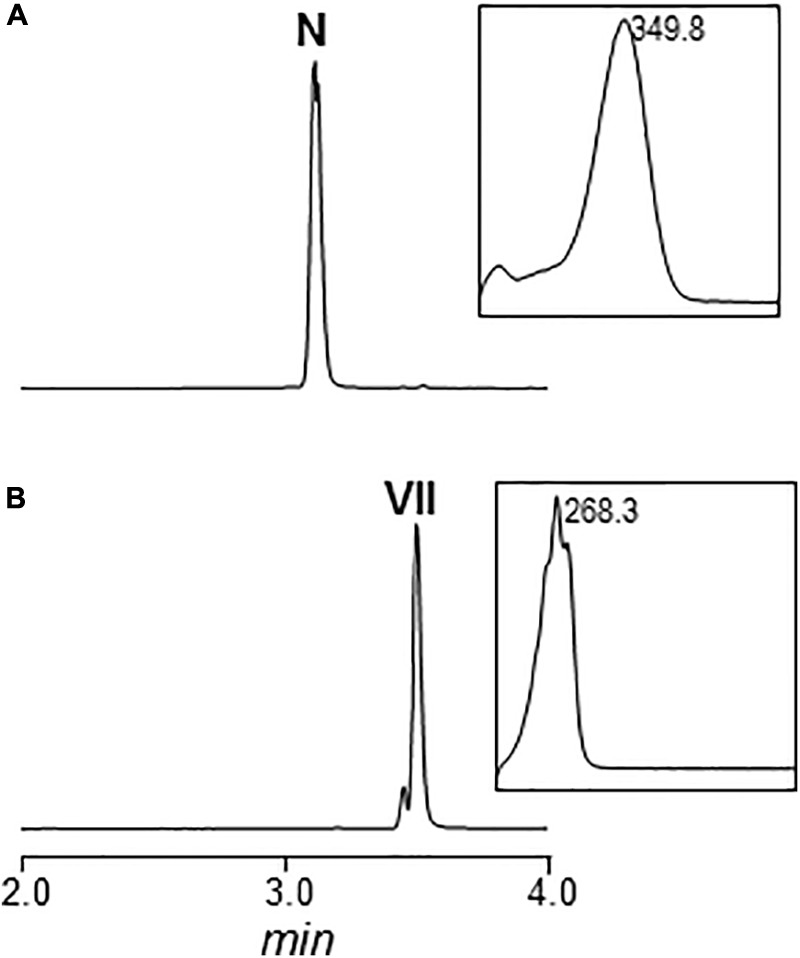
Chemical reduction of nigrifactin with sodium borohydride (NaBrH_4_). UPLC chromatogram and UV/vis spectrum of nigrifactin before **(A)** and after **(B)** the chemical reduction. N, nigrifactin; VII, argimycin PVII. Chromatograms are shown at 272 nm.

*arpHI* and *arpHII* are two small genes convergently transcribed that code for proteins belonging to the NTF2_like superfamily, which have been proposed to be cyclases (Ye et al., 2017). Using pHZMutorf9a and pHZMutorf9b, each gene was individually mutated by replacing the corresponding wild type copies by apramycin resistance cassettes, generating mutants MARPHI and MARPHII, respectively (**Table 1** and Supplementary Figures S4, S5). The metabolite profiles from both mutants were similar (**Figure 2**): they only produced nigrifactin and argimycin PIX. In addition, two new peaks, absent in the wild type strain, were detected at 272 nm (peak 1; **Figure 2B**) and 230 nm (peaks 1 and 2; **Figure 2C**), with retention times of 3.04 and 3.13 min, respectively. Peak 2 contained a compound displaying a maximum at ca. 228 nm in the UV (DAD) spectrum that was identical to the previously characterized argimycin PIX (Ye et al., 2017). After purification of this compound its molecular formula was established as C_12_H_19_NO based on the pseudomolecular ion peak [M+H]^+^ observed at 194.1541 (calcd. for C_12_H_20_NO^+^ = 194.1539). NMR analysis revealed the presence of an epoxide, which accounted for the extra oxygen and degree of unsaturation (Supplementary Figures S6, S7, S10, and Supplementary Table S4). This new compound was named argimycin PXI (**Figure 1B**). On the other hand, peak 1 contained two compounds with different masses (m/z values of 192 [M+H]^+^ and 194 [M+H]^+^). Importantly, levels of the lower-mass compound greatly decreased when cultures were incubated in the dark, which enabled the purification of the higher-mass compound by cultivating MARPHII in darkness. NMR analysis confirmed it as having a structure equal to argimycin PXI but with Z stereochemistry in the middle double bond of the side chain (Supplementary Figures S8–S10 and Table S5). This new compound was named argimycin PX (**Figure 1B**). In addition, cultures of MARPHI and MARPHII revealed another peak eluting around 3.5 min (peak 3; **Figures 2B,C**). Analysis of peak 3 by HPLC-MS showed three compounds with different masses (178 [M+H]^+^, 180 [M+H]^+^ and 230 [M+H]^+^). The lowest-mass compound (178 [M+H]^+^) differed only in two units from nigrifactin (176 [M+H]^+^), and it was hypothesized that this compound (named argimycin PVII) could be an analog of nigrifactin bearing an amino rather than an imine group. To prove this, nigrifactin was subjected to chemical reduction with sodium borohydride (NaBH_4_). Thus, after 1 h of reaction nigrifactin was completely consumed and a new compound appeared with the same retention time and mass (178 [M+H]^+^) as argimycin PVII (**Figure 4**). NMR analyses (Supplementary Figures S11, S12 and Supplementary Table S6) confirmed the structure of argimycin PVII (**Figure 1B**). This compound was previously reported by Ohno et al. (2015). To enable isolation of the other two compounds from peak 3, MARPHII was cultivated on darkness since the production of argimycin PVII greatly decreased under these conditions. Accordingly, a sample enriched in the two other compounds was purified. The compound with the highest mass, the most abundant, showed a molecular formula of C_12_H_20_ClNO based on the pseudomolecular ion peak [M+H]^+^ observed at 230.1307 (calcd. for C_12_H_21_ClNO^+^ = 230.1306). NMR elucidation revealed a chlorohydrin structurally related to the epoxide found in argimycin PXI (Supplementary Figures S10, S13, S14 and Supplementary Table S8). This new compound was named argimycin PXII (**Figure 1B**). Regarding the minor compound, the molecular formula was established as C_12_H_21_N based on the pseudomolecular ion peak [M+H]^+^ observed at 180.1748 (calcd. for C_12_H_22_N^+^ = 180.1747) that was identical to argimycin PIX (Ye et al., 2017), and NMR analyses (Supplementary Figures S13, S14 and Supplementary Table S7) confirmed that this compound, which was named argimycin PVIII (**Figure 1B**), is a geometric isomer of argimycin PIX. On the other hand, careful analysis of cultures from the wild type strain and from some other *arp* mutants showed that argimycin PVII and PVIII but not argimycin PXII, were identified in the metabolite profiles of these strains (peak 3^∗^, **Figures 2B,C**). All these results confirmed that MARPHI and MARPHII are blocked before the cyclization event that leads to the formation of the five-membered ring.

#### Mutant in *arpO*

*arpO* is divergently transcribed from *arpDHI* and *arpDHII*. It shows high similarity to several putative oxidases including amino oxidases, and contains domains present in dehydrogenases proteins. Using pHZMutorf5 most of the wild type copy of *arpO* was replaced by an apramycin resistance cassette, generating mutant MARPO (**Table 1** and Supplementary Figure S15). Analysis of the metabolite profile produced by MARPO revealed that it produced all argimycins P except argimycins PI and PII (**Figure 2**). These compounds differ from other argimycins P with two rings by containing a pyridine instead of a piperidine ring and by the presence of an *N*-acetylcysteine residue attached to the polyene chain (**Figure 1A**). Moreover, two new peaks were detected in cultures of MARPO at 276 nm (**Figure 2B**), which eluted at 2.75 and 3.15 min, respectively. The early peak contained a single compound that was too unstable to be purified. The late peak (peak 4 in **Figure 2B**) was purified and showed a maximum at 288 nm in the UV/vis spectrum. The HRMS information rendered a molecular formula of C_12_H_17_N based on the observed ion [M+H]^+^ at 176.1433 (calcd. for C_12_H_18_N^+^ = 176.1434). Such formula was identical to that of argimycin PVI. Elucidation of the structure was assisted by comparing its NMR data (Supplementary Figures S16–S18, and Supplementary Table S9) to those from argimycins PIV, PV and PVI (Ye et al., 2017). The proposed structure for this new compound, named argimycin PXIV (**Figure 1B**), was similar to that of argimycin PVI (**Figure 1A**) but differing in the position of the double bond, which was between C4–C4a in the former and between C6–C7 in the latter. These results suggest that ArpO could be a dehydrogenase involved in the oxidation of the piperidine ring leading to the pyridine ring.

#### Mutants in *arpK* and arpX Genes

ArpK is highly similar to flavin reductases and its coding gene is located upstream of *arpHI*. A mutant by gene replacement was generated in this gene (MARPK) using pHZMutorf9 (**Table 1** and Supplementary Figure S19). This mutant showed a similar metabolite profile as the wild type strain, but argimycins PI/PII and argimycin PIV were not detected (**Figures 2A,B**). This result supports the function of ArpK as regenerating flavin nucleotides that could be used by ArpO.

Downstream of *arpRII* is located *arpX* that codes for a protein of unknown function. A mutant by gene replacement was generated using pHZMutorf14 (**Table 1** and Supplementary Figure S20). Analysis of the metabolite profile of the resultant mutant MARPX showed that it produced diminished amounts of all argimycins P (**Figure 2**).

#### Co-synthesis between Mutant Strains

**FIGURE 5:**
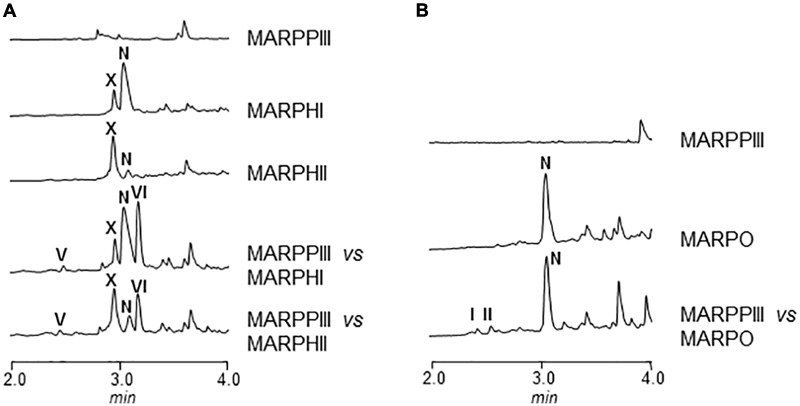
UPLC chromatograms of butanol extracts of co-synthesis products between *S. argillaceus* MARPPIII and different *arp* mutants. Chromatograms are shown at 272 nm **(A)** and 400 nm **(B)**.

In order to elucidate the order of the biosynthesis steps in argimycin P pathway, co-synthesis experiments were carried out between every mutant in *arp* structural gene and MARPPIII (PKS-minus mutant). No argimycin P containing two rings were detected when MARPN, MARPDHI, or MARPDHII were co-cultivated with MARPPIII (data not shown). These results indicated that either compounds accumulated by these mutants were not diffusible or unable to enter in the cell, or they were not real biosynthesis intermediates. However, when MARPHI or MARPHII were co-cultivated with MARPPIII, formation of argimycin PV and argimycin PVI was detected, while none of these compounds were produced by any of these mutants when grown individually (**Figure 5A**). This indicated that these mutants accumulated biosynthesis intermediates that were modified by MARPPIII leading to the formation of five-membered ring compounds. However, co-cultivation between MARPHI and MARPHII did not affect argimycin P production profiles (data not shown). These results confirm that ArpN, ArpDHI, and ArpDHII were involved in earlier biosynthesis steps, before formation of the five-membered ring, and strongly support that ArpHI and ArpHII would cooperate in the cyclization reaction leading to the formation of the five-membered ring. On the other hand, by co-cultivating MARPO with MARPPIII production of argimycin PI/PII was recovered, indicating that MARPO accumulated biosynthesis intermediates that can be modified by an *arpO* expressing strain resulting in the formation of these two argimycins P (**Figure 5B**).

**FIGURE 6:**
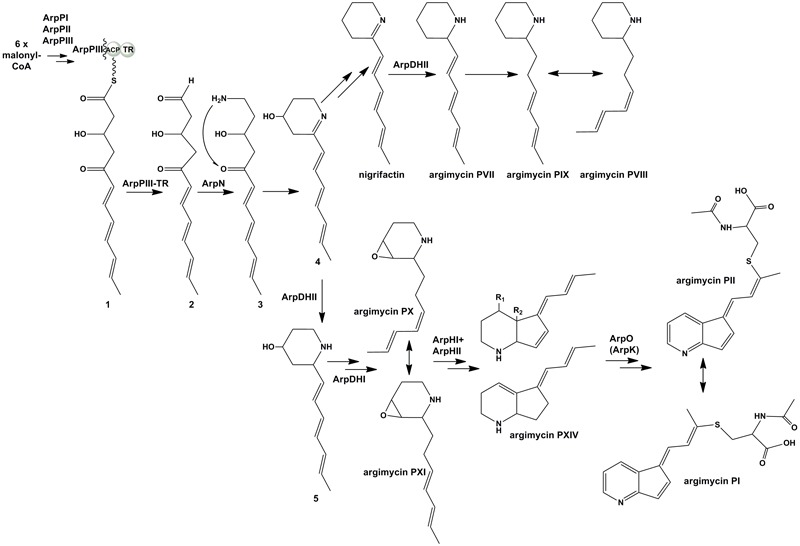
Proposed biosynthesis pathway for argimycins P. R1 = OH and R2 = OH, argimycin PIV; R1 = H and R2 = OH, argimycin PV; R1 = H and R2 = H, argimycin PVI.

Based on all these results, we propose a model for the biosynthesis of argimycins P that can be divided into several stages (**Figure 6**). In the initial steps the piperideine ring would be synthesized. The ArpPI, ArpPII, and ArpPIII PKS would render the thioester hexaketide 1 according to the specificities of the PKS catalytic domains (Supplementary Figure S21). This polyketide, similar to cyclizidine (Peng et al., 2016) and coelimycin P (Awodi et al., 2017) biosynthesis pathways, would be reductively released by the thioester reductase domain present in the PKS subunit ArpPIII to render the putative aldehyde 2. Then, transamination by aminotransferase ArpN of the aldehyde group of the released polyketide chain would occur rendering the hypothetical product 3, followed by its non-enzymatic cyclization to generate the putative biosynthesis intermediate 4 with a piperideine ring. Spontaneous dehydration and reduction of 4 would occur to provide nigrifactin.

The next step would be the formation of the piperidine ring. This is supported by the fact that all mutants in *arp* genes except MARPDHII accumulate compounds with a piperidine ring, which suggests the reduction of the imine group should be an early biosynthesis step. Most probably, this reaction would occur following the transamination reaction as it has been recently reported in the cyclizidine biosynthesis pathway (Peng et al., 2016). ArpDHII is proposed as a candidate to carry out this imine reduction step. Unlike the proposed streptazone E biosynthesis pathway (Ohno et al., 2015), neither nigrifactin nor argimycin PVII, PVIII, and PIX seem to be intermediates in the biosynthesis of argimycins P containing two rings, since recovery of its production was not achieved either in co-synthesis experiments between mutants (e.g., MARPDHI or MARPDHII vs. MARPPIII), or in bioconversion experiments with nigrifactin by mutant MARPPIII. Therefore, formation of nigrifactin and its reduction to argimycin PVII would rather form part of a biosynthesis side pathway leading to argimycin PVIII and PIX, while a different intermediate should be used in the main pathway leading to the formation of two rings-argimycins P. That intermediate could be the putative biosynthesis intermediate 4, that after reduction by ArpDHII would generate the putative intermediate 5. This compound has not been identified so far in cultures of *S. argillaceus* wild type or any *arp* mutant, however, structurally related compounds have been identified in other *Streptomyces* strains that also produce piperidine polyketide compounds (Puder et al., 2000; Groenhagen et al., 2014).

Five-membered ring cyclization would be preceded by formation of biosynthesis intermediates containing epoxy groups. This type of compounds (argimycin PX and PXI) is only produced by mutants MARPHI and MARPHII. Since these two mutants co-synthesize with mutant MARPPIII, it is deduced that compounds accumulated by these mutants are intermediates for the biosynthesis of argimycins P containing two rings. It is proposed that ArpDHI would be involved in the formation of these epoxide containing compounds by oxidizing a putative intermediate with a double bond generated by spontaneous dehydration of compound 5. The next stage would be the five-membered ring formation. According to our results and similarly to the streptazone E biosynthesis pathway (Ohno et al., 2015), this cyclization event could be coupled to ring-opening of the epoxide, and would be carried out in a cooperative manner by ArpHI and ArpHII.

The final biosynthesis step would be formation of argimycins PI/PII that contain a pyridine ring and an *N*-acetylcysteine moiety. It is proposed a role of ArpO (and ArpK) in formation of these compounds by oxidizing a biosynthesis intermediate accumulated by MARPO, including oxidation of the amino group to render the imine. This type of oxidation has already been reported in the biosynthesis of the alkaloid polyketide abikoviromycin by Tsuruoka et al. (1973), which purified an oxidoreductase from its producer that carries out the oxidation of the amino group into the imine group of this compound. Further attachment of *N*-acetylcysteine to the oxidized product synthesized by ArpO would generate argimycins PI/PII. Most probably, this would be a non-enzymatic event as it has been reported for the biosynthesis of other *N*-acetylcysteine-containing compounds (Suzuki et al., 2006; Gómez-Escribano et al., 2012; Maier et al., 2014).

### Regulation of *arp* Gene Cluster

**FIGURE 7:**
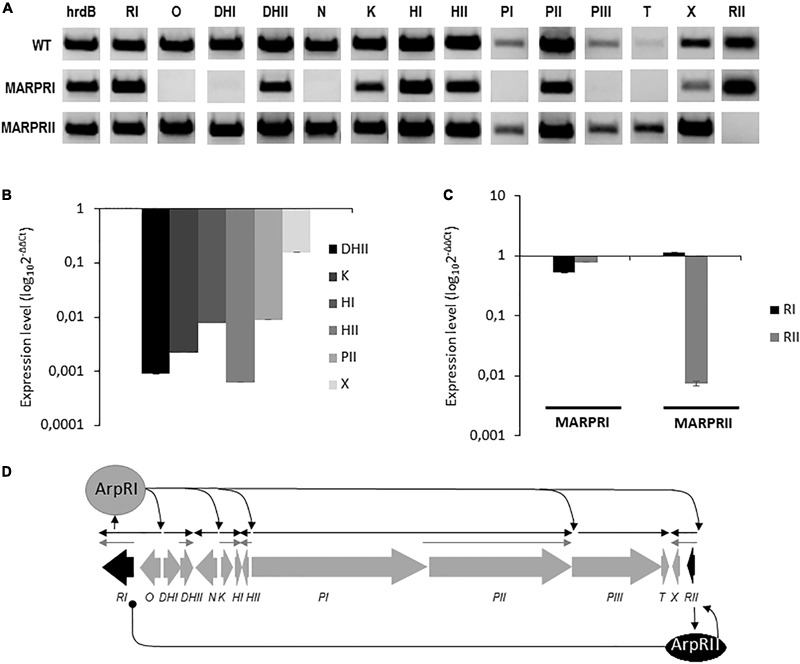
Gene expression analysis of the *arp* gene cluster. **(A)** Transcription analysis by RT-PCR of *arp* genes in the wild type (WT) and in regulatory mutants *S. argillaceus* MARPRI and MARPRII. The band intensity represents the level of transcription of each gene in the different strains. Expression of the *hrdB* gene was used to standardize the concentration of RNA. qRT-PCR quantification of the expression levels of **(B)** selected *arp* genes in MARPRI strain, and **(C)** of regulatory genes *arpRI* and *arpRII* in regulatory mutants MARPRI and MARPRII. Graphs show the relative expression of these genes in relation to the WT strain (WT), in **(B)** MARPRI, and **(C)** in MARPRI and MARPRII mutants. **(D)** Proposed model for transcriptional regulation of the *arp* gene cluster. Regulatory genes are represented in black. Black arrows indicate deduced transcriptional units ArpRI-dependent. Gray arrows indicate deduced transcriptional units ArpRI-independent. Sharp-ended and round-ended arrows indicate activation and repression, respectively.

The *arp* gene cluster contains two cluster situated regulators (CSR; Huang et al., 2005): *arpRI* codes for a SARP-like activator and its inactivation completely blocks argimycins P production; *arpRII* codes for a TetR-like repressor and its inactivation increases argimycins P production (Ye et al., 2017). To confirm their regulatory role, both genes were independently overexpressed into *S. argillaceus* and production of nigrifactin, argimycin PI/II, PV, and PVI was quantified in the resultant recombinant strains and in regulatory mutant strains MARPRI and MARPRII as well (Supplementary Figure S22). *S. argillaceus* containing the empty vector and *S. argillaceus* wild type strains were used as controls. Production of argimycins P was completely blocked in MARPRI, while it increased in MARPRII between 56 and 259%, depending of the argimycin P analyzed. On the other hand, overexpression of *arpRI* (pSETEcRI) led to an increase (between 23 and 94%), and that of *arpRII* (pSETERII) to a decrease (between 48 and 87%) in argimycins P production, depending of the argimycin P quantified. These results confirmed the role of ArpRI and ArpRII as activator and repressor in argimycin P biosynthesis, respectively. In order to delve deeper into their role in transcriptional regulation of *arp* cluster, expression analysis of *arp* genes in these mutants was evaluated by RT-PCR. Total RNA was isolated from cultures of *S. argillaceus* wild type strains and mutants MARPRI and MARPRII, after 24 h of growth. As shown in **Figure 7A**, all genes were expressed in the wild type strain including both regulatory genes. On the contrary, in MARPRI no transcripts were detected for *arpO, arpDHI, arpN, arpPI, arpPIII*, and *arpT*. Noticeable, *arpDHII, arpK, arpHI, arpHII, arpPII*, and *arpX* were still transcribed but at lower extent. This was confirmed by qRT-PCR analyses that showed the expression levels of these genes were lower in MARPRI in relation to the wild type strain (**Figure 7B**). This could be explained by the existence of alternative promoters ArpRI-independent upstream of those genes. These results were consistent with the idea that ArpRI was an essential regulator of argimycins P biosynthesis. In MARPRII mutant, all *arp* genes were transcribed apparently at higher levels than in the wild type strain. One exception was *arpRII*, since it was not transcribed (**Figure 7A**). To better understand the relationship between both regulatory genes, qRT-PCR analyses of them were carried out in each regulatory mutant. As observed in **Figure 7C**, expression of *arpRI* and *arpRII* was slightly lower in MARPRI, and expression of *arpRI* was higher and that of *arpRII* was much lower in MARPRII, in comparison to the wild type strain. Based on these results, we propose a model for the regulation of argimycins P biosynthesis (**Figure 7D**), in which ArpRII positively controls expression of its coding gene and negatively controls expression of *arpRI* whose gene product in turn positively controls expression of all *arp* genes.

## Conclusion

We have characterized the post-piperideine ring biosynthesis steps of argimycins P, a group of piperidine-containing polyketides, significantly broadening the knowledge about the biosynthesis steps of this type of compounds and of its regulation. Moreover, we have pointed out important differences between the biosynthesis pathway of argimycins P and that of the structurally related streptazone E. Thus, the *arp* cluster shows high synteny to other clusters in *Streptomyces*, such as the streptazone E gene cluster (*stz*) from *Streptomyces* sp. MSC090213JE08 (Ohno et al., 2015) (Supplementary Figure S23). Interestingly, *Streptomyces* sp. MSC090213JE08 does not produce the two ring-containing argimycins P but Streptazone E and in addition, *S. argillaceus* produce a number of argimycins P (PVII to PXII) bearing a single piperidine ring, which have not been identified in cultures of *Streptomyces* sp. MSC090213JE08. These facts suggest differences between both pathways at different levels. Thus, the hexaketides synthesized by the Arp and the Stz PKS should be different, since module 5 from ArpPIII contains an inactive dehydratase domain (Supplementary Figure S21), while the corresponding module from StzB contains an active one. Another difference could be at the regulatory level. Transcriptional analyses of the *arp* cluster have shown that some genes could be expressed from two different promoters, an ArpRI-dependent and an ArpRI-independent, which could account for higher expression of those genes and consequently of the corresponding gene products. The imine reductase *arpDHII* is one such gene and this could partially explain why most one ring-containing argimycins P so far identified in *S. argillaceus* contain a piperidine ring, while in the case of *Streptomyces* sp. MSC090213JE08 these compounds contain a piperideine ring. In addition, differences could exist at the enzymatic level. Thus, ArpO shows high similarity (80% identical aminoacids) to the FAD-dependent monooxygenase StzK identified in the streptazone E gene cluster (Ohno et al., 2015). However, StzK has been involved in the formation of the epoxide ring which is later opened in the cyclization step to form the five-membered ring, while ArpO would act after the five-membered ring was formed, oxidizing the amino group. All these differences indicate that similar gene clusters can direct the biosynthesis of structurally similar but different compounds.

On the other hand, we have identified five new compounds (argimycins PVIII, PX, PXI, PXII, and PXIV) that can be expanded to produce other analogs, and generated several mutant strains that can be used as hosts to express genes from other biosynthesis gene clusters (e.g., from other polyketide alkaloid gene clusters) and vice versa, the characterized *arp* coding genes can be expressed in other producers to generate new potentially bioactive compounds (Méndez and Salas, 2003; Olano et al., 2014). Moreover, *arp* coding enzymes such as the ArpDHII imine reductase could contribute to extend the biocatalysis toolbox with new enzymes with potential application to make pharmaceuticals and agrochemicals (Schrittwieser et al., 2015; Sigrist et al., 2015).

## Author Contributions

CM and JS conceived and designed the project. SY conducted the experiments. AB and SY carried out compound purifications. CM wrote the manuscript. JS, CO, JG-S, and FM contributed to preparing the final version of the paper. All authors read and approved the final manuscript.

## Conflict of Interest Statement

The authors declare that part of this work was included in a Spanish patent.
