# Supplementary material for: New Insights into the Biosynthesis Pathway of Polyketide Alkaloid Argimycins P in Streptomyces argillaceus
Source: Front Microbiol. 2018 Feb 16;9:252. doi: 10.3389/fmicb.2018.00252 (PMC5820336; doi:10.3389/fmicb.2018.00252)

Supplementary Material

New insights into the biosynthesis pathway of polyketide alkaloid argimycins P in *Streptomyces argillaceus*

Suhui Ye[1,2], Alfredo F. Braña[1,2], Javier González-Sabín[3], Francisco Morís[3], Carlos Olano[1,2], José A. Salas[1,2] and Carmen Méndez[1,2]*

*** Correspondence:** Carmen Méndez; cmendezf@uniovi.es

<u>Construction of plasmids for generating mutants</u>

Several plasmids were constructed using oligonucleotides described in Table S1, to generate mutants by gene replacement:

<u>pHZMutorf5</u>: a 1.95 kb DNA fragment containing *arpDHI* and the 5'-end of *arpO* was amplified using oligonucleotides Mutorf5_1_A and Mutorf5_1_B, subcloned into pCRBlunt and released as an EcoRI (this site from the vector)-HindIII fragment to be subcloned into the same site of pUO9090 upstream of the apramycin resistance cassette, generating pUOMutorf5_1. Also, a 1.99 kb DNA fragment containing the 3'-end of *arpO* and the 5'-end of *arpRI* was amplified using oligonucleotides Mutorf5_2_A and Mutorf5_2_B, subcloned into pCRBlunt and released as a BglII-NotI (this site from the vector) to be subcloned into the BamHI and NotI sites of pUOMutorf5_1, downstream of the apramycin resistance cassette, generating pUOMutorf5. Finally, the insert was rescued from this construct as a XbaI fragment and subcloned into the same site of pHZ1358.

<u>pHZMutorf6</u>: a 1.99 kb DNA fragment containing *arpO* and the 5'-end of *arpDHI* was amplified using oligonucleotides Mutorf6_1_A and Mutorf6_1_B, subcloned into pCRBlunt and released as an EcoRI (this site from the vector)-HindIII fragment to be subcloned into the same sites of pUO9090 upstream of the apramycin resistance cassette, generating pUOMutorf6_1. Also, a 1.62 kb DNA fragment containing *arpDHII* and the 3'-end of *arpDHI* was amplified using oligonucleotides Mutorf6_2_A and MutAT2_A_bis, subcloned into pCRBlunt and released as an EcoRV fragment (using one site from the vector) to be subcloned into the same site of pUOMutorf6_1 downstream of the apramycin resistance cassette. Finally, the insert was rescued from this construct as a XbaI fragment and subcloned into the same site of pHZ1358.

<u>pHZMutorf7long</u>: A 1.94 kb DNA fragment containing *arpDHI* and the 5'-ends of *arpDHII* and *arpO* was amplified using oligonucleotides Mutorf7_1_A/Mutorf5_1_A, subcloned into pCRBlunt and released as an EcoRI fragment (using both sites from the vector) to be subcloned into the same site of pUO9090, upstream of the apramycin resistance cassette, generating pUOMutorf7_1. Also, a 3.86 kb DNA fragment containing *arpN*, *arpK*, *arpHI* and the 3'-ends of *arpDHII* and *arpHII* was amplified using oligonucleotides Mutorf7_2_A and Mutorf9b_2_A, subcloned into pCRBlunt and released as an EcoRV fragment to be subcloned into the same site of pUOMutorf7_1, downstream of the apramycin resistance cassette. Finally, the insert was rescued from this construct as a SpeI fragment and subcloned into the XbaI site of pHZ1358.

<u>pHZMutorf9</u>: A 1.7 kb DNA fragment containing the 5'-ends of *arpN* and *arpK*, was amplified using oligonucleotides Mutorf9_1_A/Mutorf9_1_B, subcloned as a BamHI-HindIII fragment into the BglII and HindIII sites of pUO9090, upstream of the apramycin resistance cassette, generating pUOMutorf9_1. Also, a 2.03 kb DNA fragment containing *arpHI*, *arpHII*, the 5'-end of *arpPI* and the 3'-end of *arpK*, was amplified using oligonucleotides Mutorf9_2_A/Mutorf9_2_B, subcloned as a BamHI-XbaI fragment into de same sites of pUOMutorf9_1, downstream of the apramycin resistance cassette, generating pUOMutorf9. Finally, the insert was rescued from this construct as a SpeI fragment, and subcloned into de XbaI site of pHZ1358.

<u>pHZMutorf9a</u>: A 1.93 kb DNA fragment containing *arpK* and the 5'-ends of *arpN* and *arpHI*, was amplified using oligonucleotides Mutorf9a_1_A/Mutorf9a_1_B, and subcloned as a BglII-HindIII fragment into the same sites of pUO9090, upstream of the apramycin resistance cassette, generating

pUOMutorf9a_1. Also, a 1.99 kb DNA fragment containing *arpHII*, the 5'-end of *arpPI* and the 3'-end of *arpHI*, was amplified using oligonucleotides Mutorf9a_2_A/Mutorf9a_2_B, and subcloned as an EcoRV-XbaI fragment into the same sites of pUOMutorf9a_1, downstream of the apramycin resistance cassette. Finally, the insert in the resultant construct pUOMutorf9a was rescued as a SpeI fragment and subcloned into the XbaI site of pHZ1358.

pHZMutorf9b: A 1.78 kb DNA fragment containing the 5'-ends of *arpHII* and *arpPI* was amplified using oligonucleotides Mutorf9b_1_A/Mutorf9b_1_B, and subcloned as a HindIII-PstI fragment into the same sites of pUO9090, upstream of the apramycin resistance cassette, generating pUOMutorf9b_1. Also, a 1.59 kb DNA fragment containing *arpK*, *arpHI*, the 5'-end of *arpN* and the 3'-end of *arpHII*, was amplified using oligonucleotides Mutorf9b_2_A/Mutorf9b_2_B, and subcloned as an EcoRV-XbaI fragment into the same sites of pUOMutorf9b_1, downstream of the apramycin resistance cassette. Finally, the insert from the resultant construct pUOMutorf9b was rescued as a SpeI fragment and subcloned into the XbaI site of pHZ1358.

pHZMutorf14: A 1.85 kb DNA fragment containing *arpRII* and the 5'-ends of *arpX* and *orf4*, was amplified using oligonucleotides Mutorf17_1_A/Mutorf17_1_B, and subcloned as a BglII fragment in the same site of pUO9090, in the right orientation upstream of the apramycin resistance cassette, generating pUOMutorf14_1. Also, a 1.53 kb DNA fragment containing *arpT* and the 3'-ends of *arpX* and *arpPIII*, was amplified using oligonucleotides Mutorf14_2_A/Mutorf14_2_B, subcloned into pCRBlunt and released as an EcoRV-XbaI fragment to be subcloned into the same sites of pUOMutorf14_1. Finally, the insert from the resultant construct pUOMutorf14 was rescued as a SpeI fragment and subcloned into the XbaI site of pHZ1358.

Table S1: oligonucleotides used for PCR to generate and to verify mutants.

PRIMER	SEQUENCE 5'-3'
**PRIMERS DESIGNED TO GENERATE MUTANTS**	
**Mutorf6_1_A**	AAATCTAGAGTTCTCCCGGGGAAGTGA
**Mutorf6_1_B**	AAAAAGCTTTCTCCAGGAGGTCGATGT
**Mutorf6_2_A**	TTTCATATGTTCTACCGGGACCTGCA
**MutAT2_A_bis**	AAAGATATCCTGATGCTCGGTCTGGAGTT
**Mutorf7_1_A**	TTTTCTAGATTGCCCTTCGCCTTGAGC
**Mutorf7_2_A**	AATGATATCGGCATCAACAGCGACTTCTC
**Murorf9a_1_A**	ATTAGATCTGCTCTTGGCGAGGGTGTA
**Murorf9a_1_B**	AAAAAGCTTCGGGTCATCGGTGAGTTC
**Murorf9a_2_A**	AAAGATATCGAGGACGGTGAGCTGGTC
**Murorf9a_2_B**	AAATCTAGAGTGGCCGATGTTGGTCTT
**Mutorf9b_1_A**	AAAAAGCTTGCCGGTGACGATCCAC
**Mutorf9b_1_B**	AGGCTGCAGTGAAGGCGGCGACATAAC
**Mutorf9b_2_A**	AAAGATATCCTGTACGCCGTCGACGAC
**Mutorf9b_2_B**	AAATCTAGAATCGCGGGGTTGTTGTG
**Mutorf5_1_A**	ATGTCTAGATTGTCAGCGGAACGGTTC
**Mutorf5_1_B**	TTTAAGCTTTTGCCCTTCGCCTTGAGC
**Mutorf5_2_A**	AAAAGATCTCAAGGTGCTCTCCGTCAAG
**Mutorf5_2_B**	AAATCTAGAGCAGTGAGAAGACGACCGTA
**Mutorf9_1_A**	AAAGGATCCGACCAGGTAGCCGAACA
**Mutorf9_1_B**	AAGAAGCTTGTGGCGAACTGGGACATC
**Mutorf9_2_A**	AAAGGATCCGCGTTGACGTTCTTCGAC
**Mutorf9_2_B**	AGATCTAGAGAAGCGGGACTCGGTGAC
**Mutorf17_1_B**	AAAAGATCTATGATCGCGTCCGCTTC
**Mutorf17_1_A**	TTTAGATCTCGGGGTGCTGACTGATCT
**Mutorf14_2_A**	AAAGATATCCACGCCTTCACCTTCCAC
**Mutorf14_2_B**	AAATCTAGACATGACCTTCGCCGAGTT
**PRIMERS DESIGNED TO VERIFY MUTANTS**	

**Mutorf5_1_A**	ATGTCTAGATTGTCAGCGGAACGGTTC
**Mutorf5_1_B**	TTTAAGCTTTTGCCCTTCGCCTTGAGC
**Orf7_A bis**	GGGACTAGTAATGAGGAGCAAGTCAATGT
**Orf7_B bis**	AAATCTAGAATCAGCCCTGCTTGAACGC
**Mutorf9_2_A**	AAAGGATCCGCGTTGACGTTCTTCGAC
**Mutorf9b_2_A**	AAAGATATCCTGTACGCCGTCGACGAC
**Murorf9a_2_A**	AAAGATATCGAGGACGGTGAGCTGGTC
**MutAT1_A**	ATTGGATCCTTCGGCGTAGTTGTCACC
**MutOrf5C_A**	TTTACGCCGGGAACACTG
**MutOrf5C_B**	GGGGAGGGCGATTATCA
**MutAT1_B**	AAAAAGCTTGGGTGCAGGGAGAACT
**Mutorf9a_1_B**	AAAAAGCTTCGGGTCATCGGTGAGTTC
**MutTetR_2_A**	GGATCCTACGGGGTCAACCTGCGCTC
**MutTetR_2_B**	GATATCCTGGACGACCCCGAACTGCT

Table S2: oligonucleotides used for PCR to express genes

PRIMER	SEQUENCE 5'-3'	GENE
**Orf6_A bis**	AATGCTAGCCCTAGGAAATCACCAAGGAGCT	*arpDHI*
**Orf6_B bis**	GGGTCTAGAGGCGGCGTATTACAGGAA	
**Orf7_A bis**	GGGACTAGTAATGAGGAGCAAGTCAATGT	*arpDHII*
**Orf7_B bis**	AAATCTAGAATCAGCCCTGCTTGAACGC	
**Orf9a_A bis**	TTTACTAGTACGAAGGAGATGCTCATG	*arpHI*
**Orf9a_B bis**	GGGTCTAGAGTTCAGACGCTGATGTC	
**Orf9b_A bis**	GGGACTAGTGAGGACAGAAGAATGTCG	*arpHII*
**Orf9b_B bis**	AAATCTAGAATCCGTGGTGTCAGTCGC	
**Orf9_A bis**	GCCACTAGTCCGAAGAGGGGAAATGACT	*arpK*
**Orf9_B bis**	TTTTCTAGAATGGCGTGCGGAGTCGTCA	
**Orf5_A bis**	TGGACTAGTTTGTGTACGAGGGGACTGGG	*arpO*
**Del59b_B**	GGGTCTAGAATCCGTTCGGGGTCAGCT	

**SARP_ATG_Abisbis**	CCATGCTGAGGGACTATGTG	*arpRI*
**378SARP_B**	TTCCGAGTGGCGTGCC	
**SA1701orf4A**	AAAGGATCCACCCCGCGAAAGCTGTGT	*arpRII*
**SA1701orf4B**	AATGGATCCTCCTCACCTTCGACGGCG	

Table S3: oligonucleotides used for PCR to study gene expression.

PRIMER	SEQUENCE 5’-3’	GENE
**PRIMERS DESIGNED FOR RT-PCR**		
**RT hrdB A**	CTCGAGGAAGAGGGTGTGAC	*hrdB*
**RT hrdB B**	GCCGATCTGCTTGAGGTAGT	
**RTorf4_A**	ACCAGACGGTACCCATCAGT	*arpRI*
**RTorf4_B**	CACAGCACGCCGACTTC	
**RTorf5_A**	GAGGCGAAGTTGATGGAGTT	*arpO*
**RTorf5_B**	CGCGGTAGTACTCCAGGTTC	
**RTorf6_A**	GCTTCAACTCCGGTGTCC	*arpDHI*
**RTorf6_B**	GGAGGAAGCCGAGGTAGG	
**RTorf7_A**	CCTACGAGGTGCTGAACGAC	*arpDHII*
**RTorf7_B**	AGGCTGGAGATCTCGGTGT	
**RTorf8_A**	GACCAGGTAGCCGAACAGTC	*arpN*
**RTorf8_B**	GAATTCGTCCGGGAGGTC	
**RTorf9_A**	ACCACATCCACGCCATGA	*arpK*
**RTorf9_B**	AACTTCCCGTCGAAGAACG	
**RTorf9a_A**	CGACCTGTACAGCCTGGAAC	*arpHI*
**RTorf9a_B**	AGCTGCCCTTGACCTGGAC	
**RTorf9b_A**	CTCGCGGTGTTGATCTCC	*arpHII*

**RTorf9b_B**	GCGCACCTCGAGAGTTATGT	
**RTorf10_A**	TGACCCACCACGGACTG	*arpPI*
**RTorf10_B**	AGTAGTCGGGCGAGGTGAG	
**RTorf11_A**	GTTGATTCCGTGGGTGTTGT	*arpPII*
**RTorf11_B**	GAGTGGATGAGGCGGTAGAG	
**1701 RTA**	CTCGTCCTGGAGGTGGTG	*arpPIII*
**1701 RTB**	CGCGGTAGTACTCCAGGTTC	
**RTorf13_A**	GACCTCCCTGACCCATCTG	*arpT*
**RTorf13_B**	GGGTGTCGAGGTAGAAGTGG	
**RTorf14_A**	ACGAGTCGTGGAAGGTGAAG	*arpX*
**RTorf14_B**	CGTCTACCGGGAGATCAGTC	
**RTorf16_A**	TGTCCGACATCAAGCACTTC	*arpRII*
**RTorf16_B**	GTCACCACGTCCTGGATACC	
**PRIMERS DESIGNED FOR QPCR**		
**qhrdB A**	TCTGTCATGGCGCTCATTGA	*hrdB*
**qhrdB B**	CGCAGTACGTTTTTCCACTGAGT	
**qRTSARP_A bis**	CGCAGGCTCCAGATCGAGTT	*arpRI*
**qRTSARP_B bis**	CTCCTCCACCCCAACCAGAC	
**qRTTetR_A**	CGTGGTACGGCTCTTCTGGC	*arpRII*
**qRTTetR_B**	CGAAGGTGGAGTAGAGGCTGG	
**qRTorf7_Abis**	CACCGCCTTTGTGAAGAACG	*arpDHII*
**qRTorf7_Bbis**	CTCGATCGCCTGCTTGATGT	
**qRTorf9_A**	ACTTCGCTGCGGGATGTGAT	*arpK*
**qRTorf9_B**	GTCATGGCGTGGATGTGGTC	
**qRTorf9a_A**	ACTCCTACACCGCCGACGAC	*arpHI*
**qRTorf9a_B**	ATCAGCCACTTGCCGTCCTC	
**qRTorf9b_A**	CTCACCGGATTGCCCTTCTC	*arpHII*

**qRTorf9b_B**	CGGCGCACCTCGAGAGTTAT	
**qRTPKSII_Abis**	CACCGCCTACTCGACCCTCT	*arpPII*
**qRTPKSII_Bbis**	GCTCGTCGGCCAGATATTCC	
**qRTorf14_A**	TCCGGGGTGCTGACTGATCT	*arpX*
**qRTorf14_B**	ACCTTCGGCGTCCTGACATC	

Table S4: NMR data for argimycin PXI (CD_3OD, 500/125 MHz)

Carbon	δ_H (mult, J in Hz)	δ_C (ppm)
2	3.14 (ddd, 12.6, 5.7, 3.3)<br>2.96 (ddd, 12.7, 10.9, 4.8)	36.3
3	2.30 (m)<br>2.24 (m)	20.9
4	3.44 (br m)	49.2
5	3.25 (br d, 3.9)	50.6
6	3.50 (br t, 7.50)	53.3
7	1.97 (m)<br>1.84 (m)	29.8
8	2.31 (m)	27.6
9	5.56 (ddd, 14.0, 7.0	127.7
10	6.14 (dd, ca. 14.2, 10.8)	132.2
11	6.05 (dd, 14.0, 11.0)	130.9
12	5.65 (dq, 13.8, 6.8)	127.7
13	1.73 (br d, 6.7)	16.6

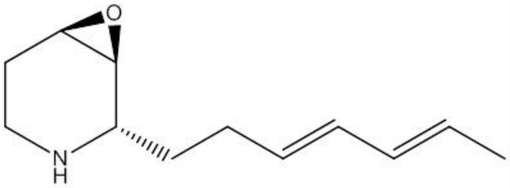

Table S5: NMR data for argimycin PX (CD_3OD, 500/125 MHz)

Carbon	δ_H (mult, J in Hz)	δ_C (ppm)
2	3.15 (ddd, 12.7, 5.7, 3.3)<br>2.96 (ddd, 12.7, 10.9, 4.7)	36.3
3	2.31 (m)<br>2.25 (m)	21.0
4	3.44 (br m)	49.1
5	3.29 (br d, 4.1)	50.6
6	3.50 (br t, 7.50)	53.4
7	1.97 (m)<br>1.83 (m)	30.0
8	2.42 (m)	22.8
9	5.31 (m)	125.3
10	6.08 (t, 10.9)	130.5
11	6.41 (brt, 13.0)	126.2
12	5.77 (dq, 13.6, 6.8)	130.3
13	1.80 (br d, 6.7)	16.9

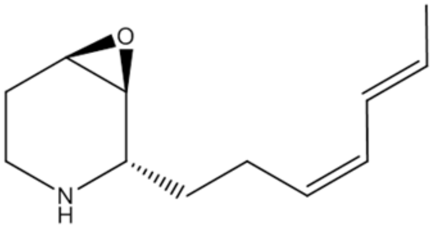

Table S6: NMR data for argimycin PVII (CD_3OD, 500/125 MHz)

Carbon	δ_H (mult, *J* in Hz)	δ_C (ppm)
2	3.09 (br dt, 12.3)<br>3.00 (tt, 12.0, 3.5)	45.6
3	1.65 (m)<br>1.49 (m)	24.6
4	1.84 (m)<br>1.48 (m)	23.7
5	1.74 (m)<br>1.34(m)	31.3
6	3.22 (m)	58.3
7	5.62 (dd, 15.0, 6.9)	133.7
8	6.24 (dd, overlap)	130.5
9	6.09 (ddd, overlap)	129.5
10	6.20 (dd, ca. 14.0, 12.0)	133.1
11	6.09 (dd, overlap)	131.4
12	5.74 (dq, ca. 14.0, 6.8)	129.4
13	1.77 (br d, 6.8)	16.9

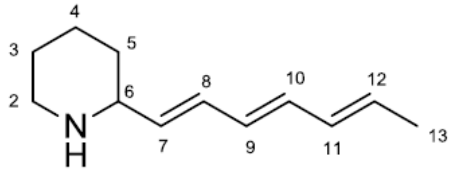

Table S7: NMR data for argimycin PVIII (CD_3OD, 500/125 MHz)

Carbon	δ_H (mult, *J* in Hz)	δ_C (ppm)
2	3.37 (br dt, 13.0)<br>3.00 (td, 12.9, 2.9)	44.4
3	1.90 (m)<br>1.66 (m)	21.9
4	1.90 (m)<br>1.56 (qt, 12.9, 3.2)	21.6
5	2.07 (br dt, 14.0)<br>1.43 (br dd, 13.0, 2.9)	28.0
6	3.08 (m)	56.2
7	1.69 (m)	33.2
8	2.30 (m)	22.6
9	5.26 (br dd, 9.8, 7.9)	125.9
10	6.03 (m)	130.0
11	6.41 (m)	126.2
12	5.75 (dq, 13.6, 6.8)	129.8
13	1.79 (br d, 6.7)	16.9

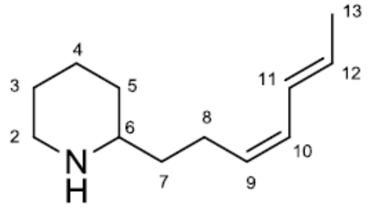

Table S8: NMR data for argimycin PXII (CD_3OD, 500/125 MHz)

Carbon	δ_H (mult, *J* in Hz)	δ_C (ppm)
2	3.33(m))<br>3.22 (br dd, 12.8, 3.4)	38.8
3	2.62 (m)<br>1.98 (br tt, 14.3, 3.7)	24.3
4	4.34 (br dd, 5.3, 2.6)	55.1
5	3.98 (br d, 3.0)	66.0
6	3.57 (ddd, 9.2, 5.3, 1.1)	53.1
7	1.90 (m)<br>1.66 (m)	28.2
8	2.22 (m)<br>2.16 (m)	27.0
9	5.53 (ddd, 14.0, 7.0)	128.1
10	6.09 (dd, ca. 14.0, 10.3)	132.0
11	6.05 (m)	131.0
12	5.64 (dq, 13.8, 6.8)	127.4
13	1.74 (br d, 6.7)	16.6

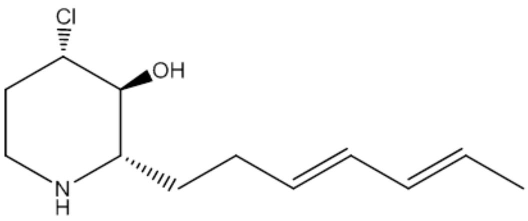

Table S9: NMR data for argimycin PXIV (CD_3OD, 500/125 MHz)

Carbono	δ_H (mult, *J* en Hz)	δ_C (ppm)
2	3.58 (br dd, 12.6, 7.1)<br>3.23 (br td, 12.3, 6.1)	41.4
3	2.61 (m)<br>2.50 (m)	21.8
4	6.11 (m)	113.6
4a	-	133.2
5	-	137.3
6	2.78 (m)<br>2.40 (m)	26.0
7	2.37 (m)<br>1.61 (m)	27.9
7a	3.99 (m)	57.0
8	6.47 (br d, 10.9)	121.0
9	6.25(ddq, 14, 11.0, 1.6)	128.2
10	5.83 (dq, 14, 6.8)	130.7
11	1.83 (brd, 6.7)	17.3

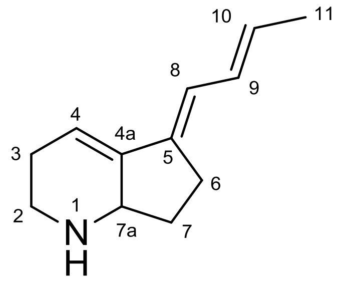

Figure S1: UPLC chromatograms of butanol extracts of complemented *S. argillaceus arp* mutants. Chromatograms are shown at 400 nm (A), 272 nm (B) and 230 nm (C). Peaks corresponding to the different argimycins P are indicated as follows: argimycins PI and PII (**I** and **II**); nigrifactin (**N**); argimycin PIV (**IV**); argimycin PV (**V**); argimycin PVI (**VI**); and argimycin PIX (**IX**).

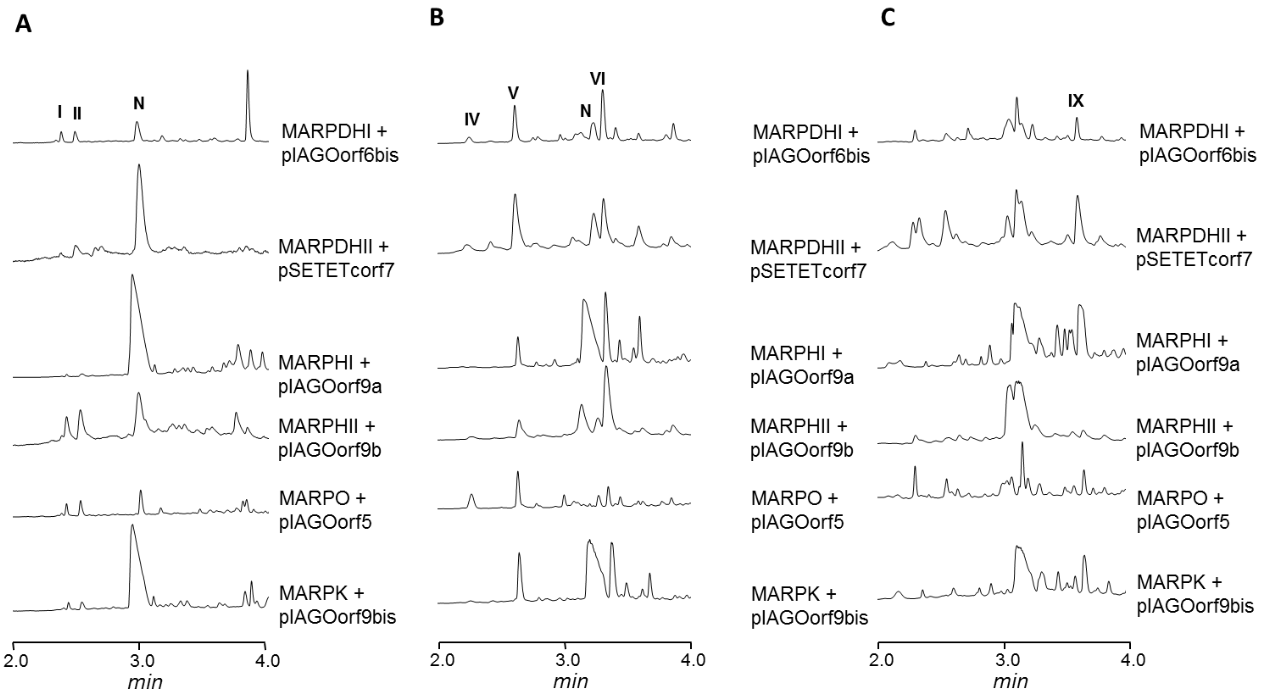

Figure S2: Generation of mutant MARPDHI. (A) Scheme representing the replacement event for generation of mutant MARPDHI. WT, wild type strain; *aac(3)IV*, apramycin resistance gene; blue rectangles in pHZMutorf6 represent DNA regions flanking the apramycin resistance cassette, which include those genes highlighted within dotted rectangles in the chromosomal DNA of the WT strain (B) PCR analysis of MARPDHI mutant. PCR products from the WT strain and from MARPDHI mutant (lanes 1-3), using oligonucleotides Mutorf5_1_A/Mutorf5_1_B. λ, PstI-digested Lambda DNA

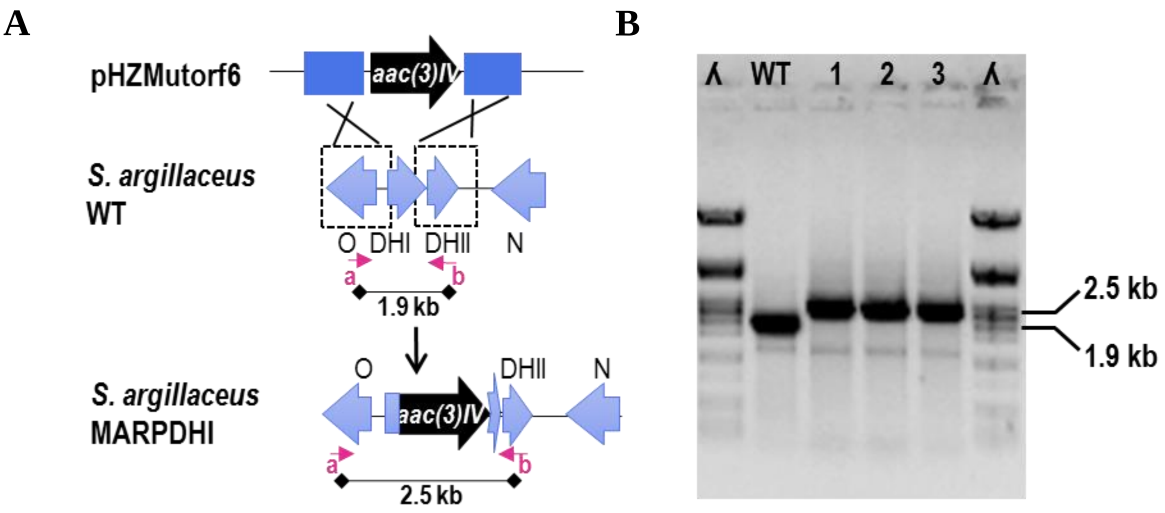

Figure S3: Generation of mutant MARPDHII. (A) Scheme representing the replacement event for generation of mutant MARPDHII. WT, wild type strain; *aac(3)IV*, apramycin resistance gene; blue rectangles in pHZMutorf7long represent DNA regions flanking the apramycin resistance cassette, which include those genes highlighted within dotted rectangles in the chromosomal DNA of the WT strain (B) PCR analysis of MARPDHII mutant. PCR products from the WT strain and from MARPDHII mutant (lanes 1, 2), using oligonucleotides Orf7_A bis/Orf7_B bis. λ, PstI-digested Lambda DNA.

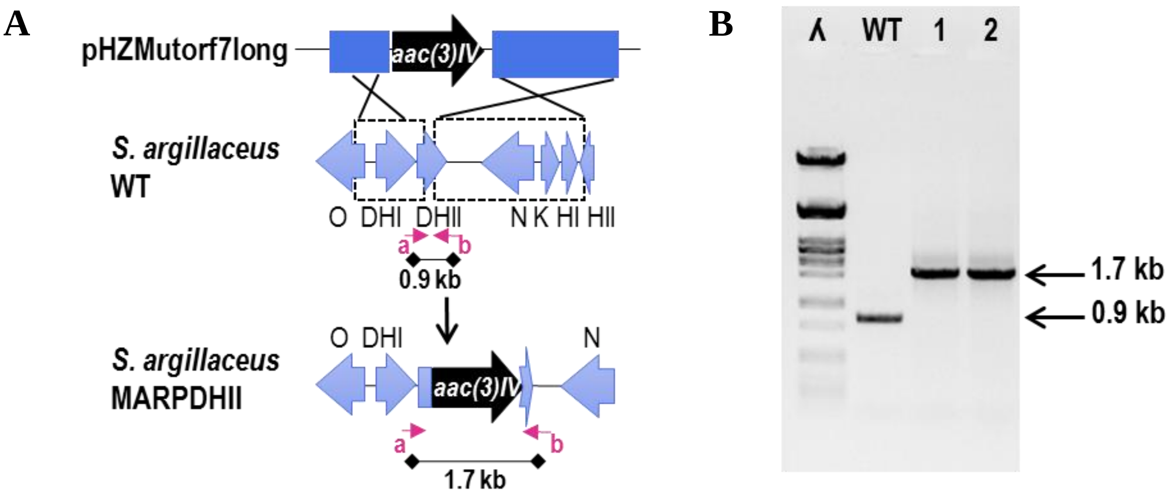

Figure S4: Generation of mutant MARPHI. (A) Scheme representing the replacement event for generation of mutant MARPHI. WT, wild type strain; *aac(3)IV*, apramycin resistance gene; blue rectangles in pHZMutorf9a represent DNA regions flanking the apramycin resistance cassette, which include those genes highlighted within dotted rectangles in the chromosomal DNA of the WT strain (B) PCR analysis of MARPHI mutant. PCR products from the WT strain and from MARPHI mutant (lanes 1-3), using oligonucleotides Mutorf9_2_A/Mutorf9b_2_A. λ, PstI-digested Lambda DNA

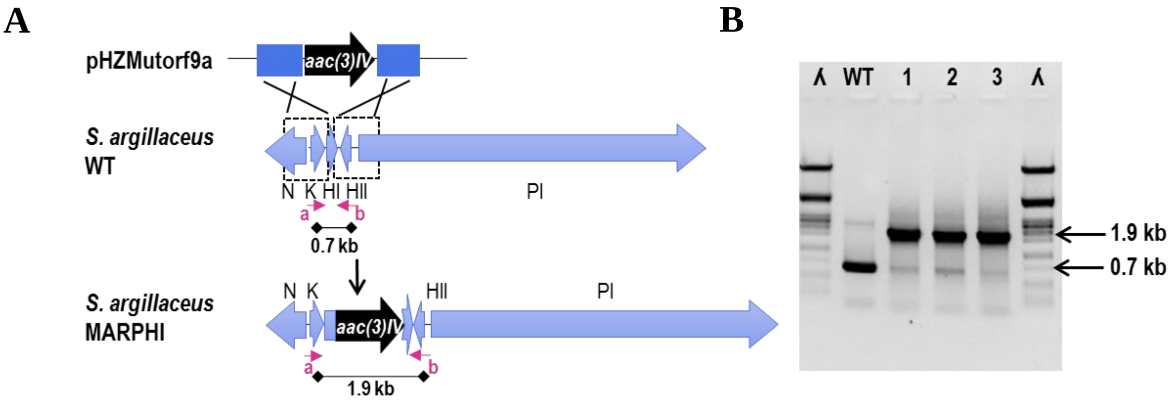

Figure S5: Generation of mutant MARPHII. (A) Scheme representing the replacement event for generation of mutant MARPHII. WT, wild type strain; *aac(3)IV*, apramycin resistance gene; blue rectangles in pHZMutorf9b represent DNA regions flanking the apramycin resistance cassette, which include those genes highlighted within dotted rectangles in the chromosomal DNA of the WT strain (B) PCR analysis of MARPHII mutant. PCR products from the WT strain and from MARPHII mutant (lanes 1-4), using oligonucleotides Mutorf9a_2_A/MutAT1_A. λ, PstI-digested Lambda DNA

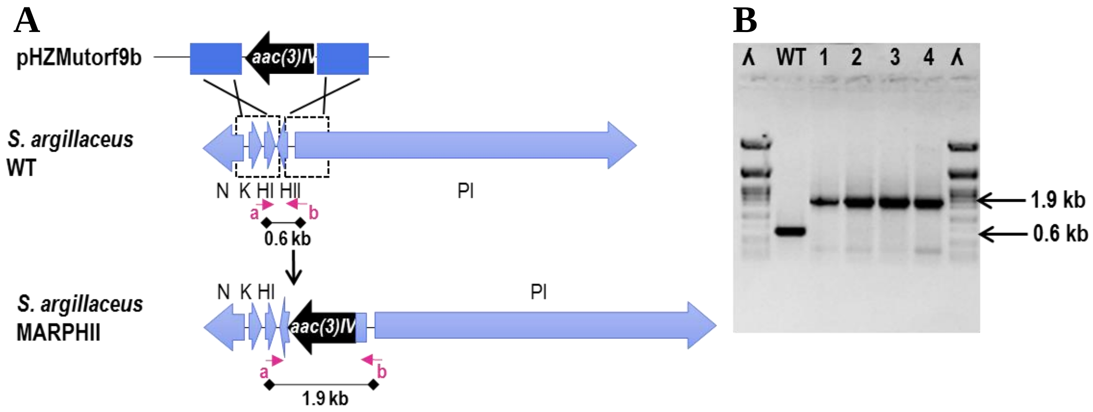

Figure S6: ^{1}H-NMR spectrum of argimycin PXI (CD_3OD, 500 MHz). **A**: overall chart. **B**: expansion of the olefinic region. **C**: expansion of the aliphatic region.

A

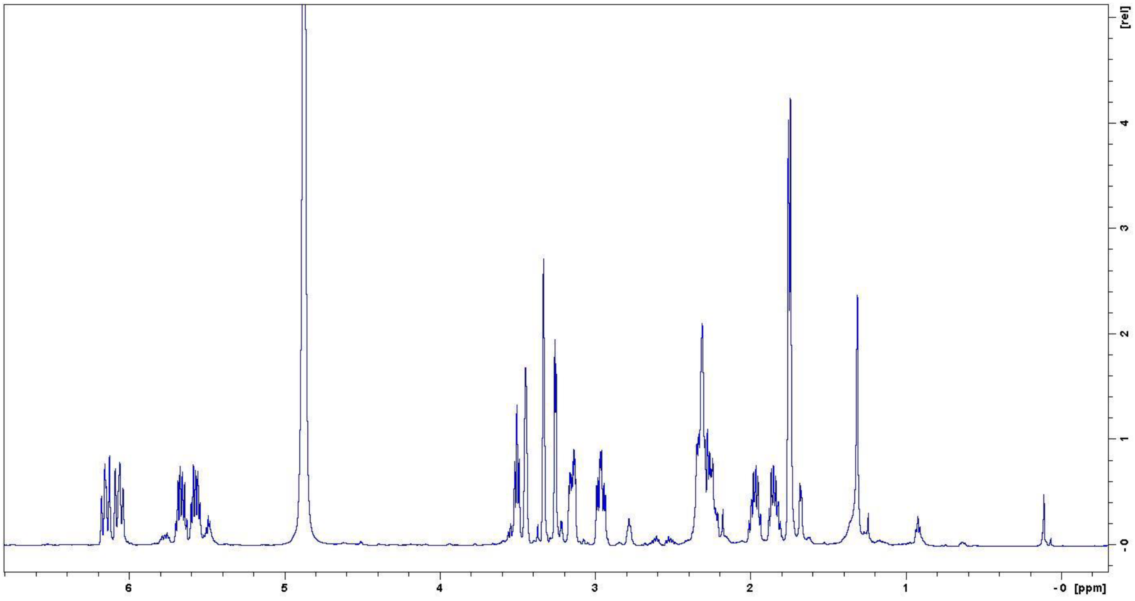

B

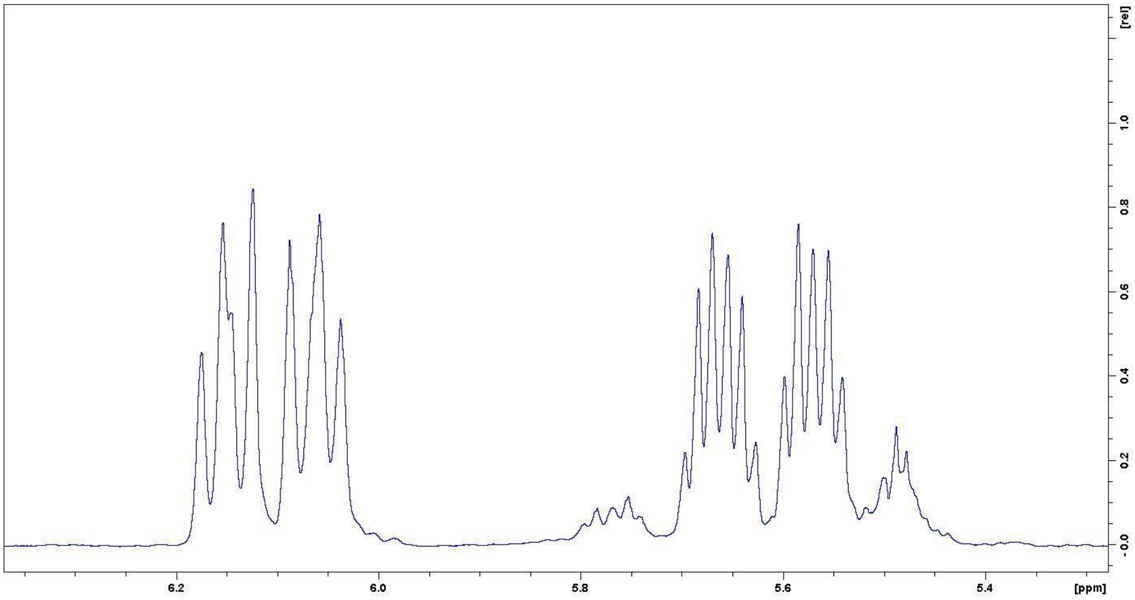

C

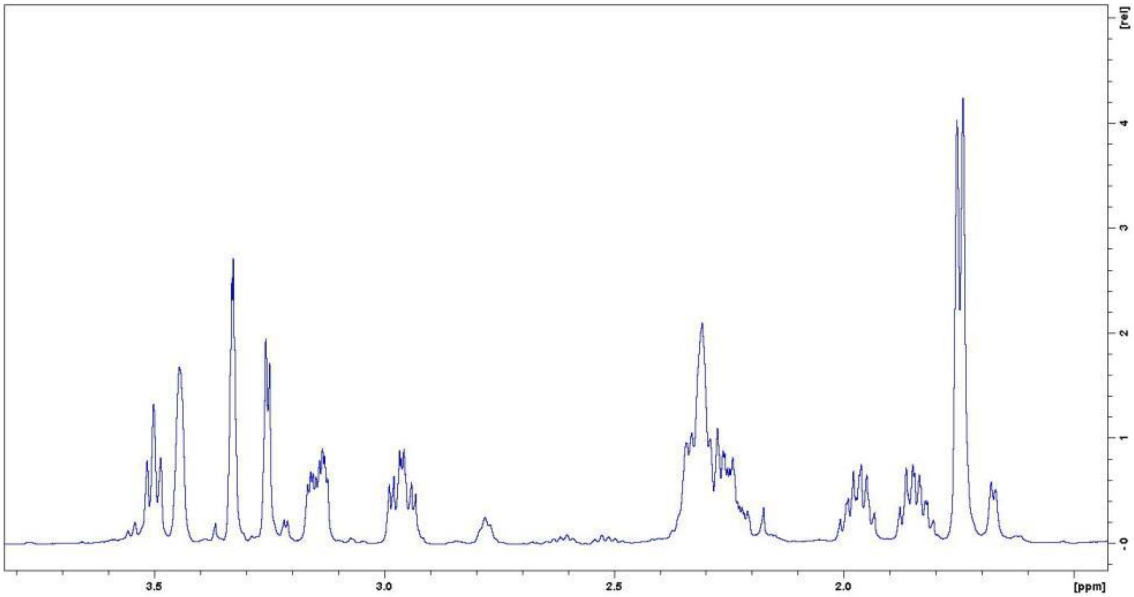

Figure S7: HSQC spectrum of argimycin PXI

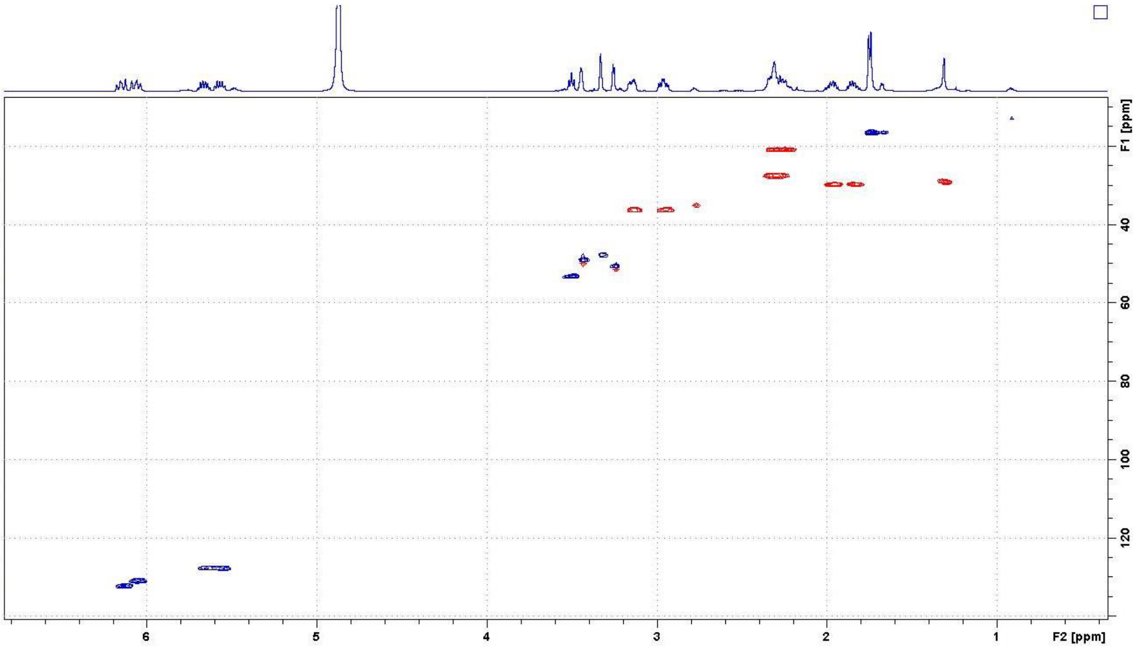

Figure S8: ^{1}H-NMR spectrum of argimycin PX (CD_3OD, 500 MHz). **A**: overall chart. **B**: expansion of the olefinic region. **C**: expansion of the aliphatic region.

A

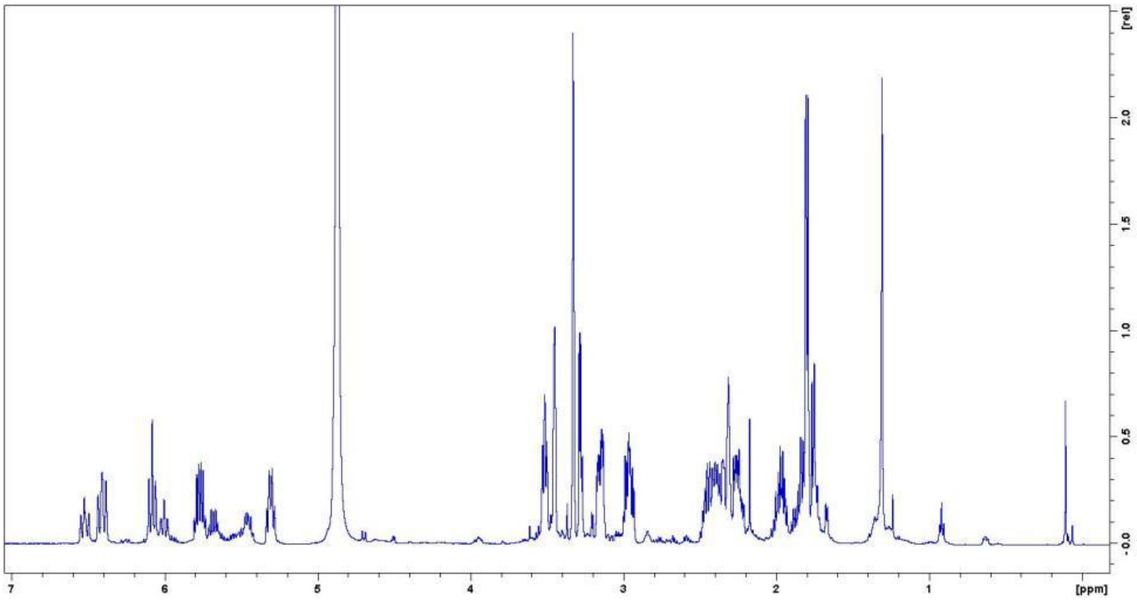

B

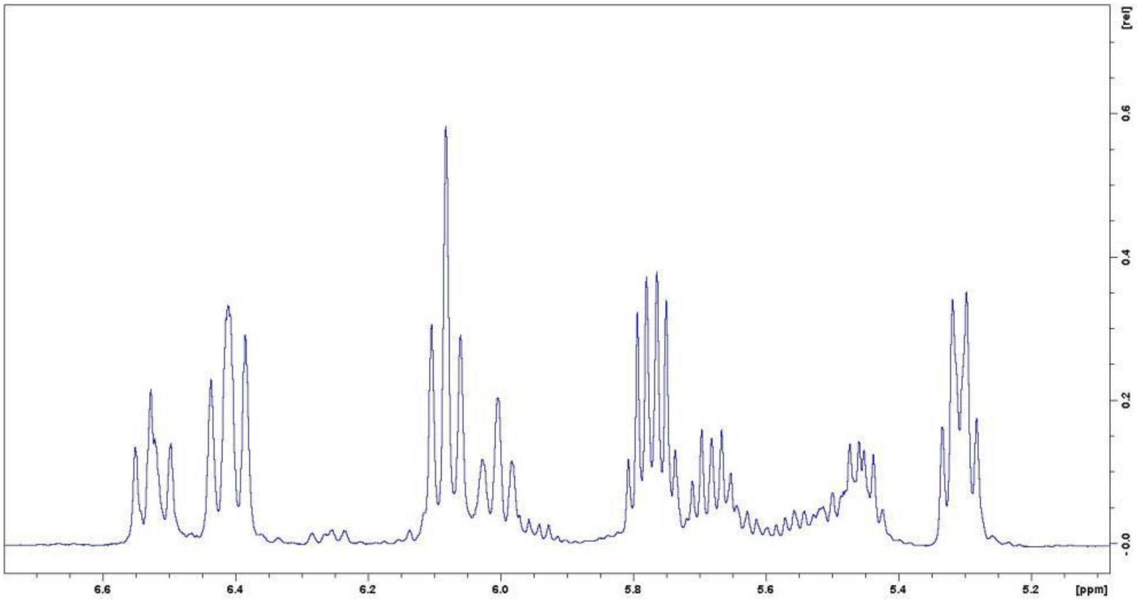

C

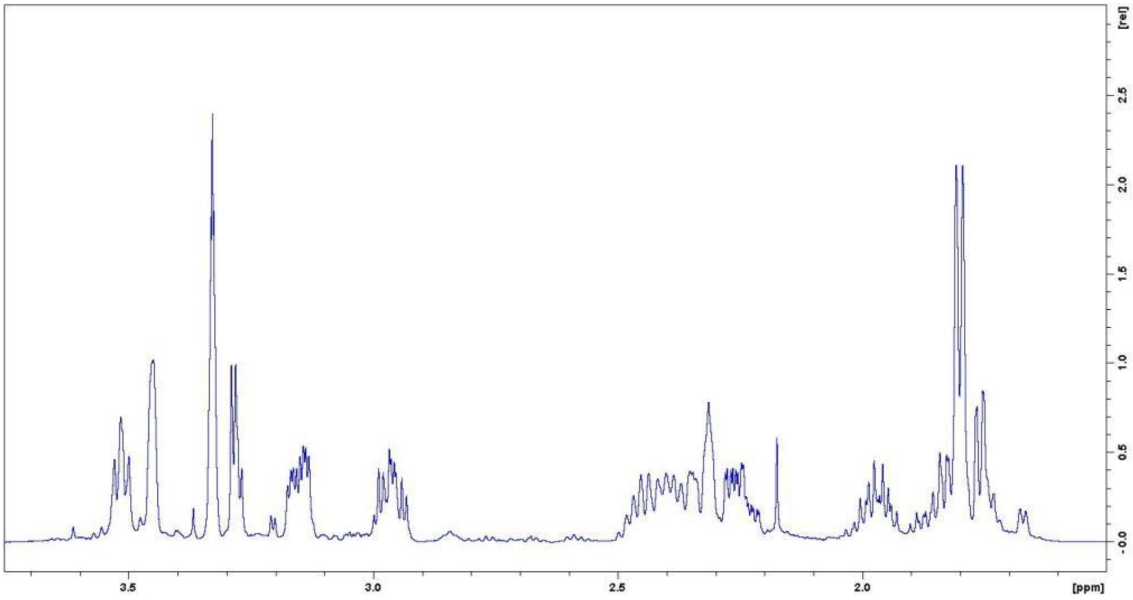

Figure S9: HSQC spectrum of argimycin PX

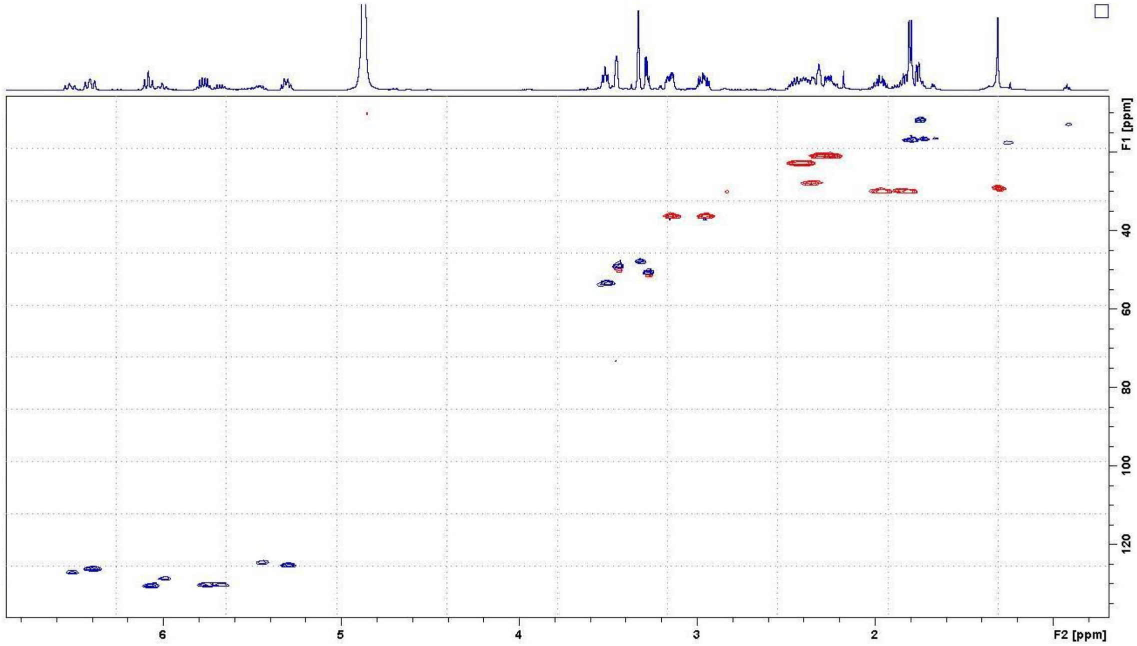

Figure S10: Relative **s**tereochemistry of argimycins PI, PXI y PXII. Regarding the relative stereochemistry of argimycin PXI, similarly to that observed for compound 6 obtained by chemical reduction of an intermediate in the biosynthesis of streptazone E (Figure S10; Ohno *et al*., 2015), we could establish a *trans* configuration between the epoxide and the side chain. H-6 appears as a broad triplet with a coupling constant of 7.50 Hz to both H-7 protons and a small, not measurable, coupling constant to H-5, indicating a dihedral angle close to 90º between H-5 and H-6. The same consideration is also valid to establish the relative stereochemistry of argimycin PX. Based on the relative stereochemistry proposed for argimycin PX, an all *trans* relative stereochemistry was proposed for the chiral centers of this compound. A small coupling constant between H-5 and H-6 of 1.1 Hz confirmed the existence of a dihedral angle close to 90º between both protons and the coupling constant of 3 Hz between H-4 and H-5 (measured in the signal for H-5) indicated a *trans* di-equatorial configuration.

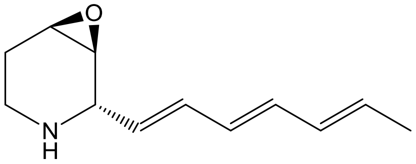

compound 6 (Ohno et al., 2015)

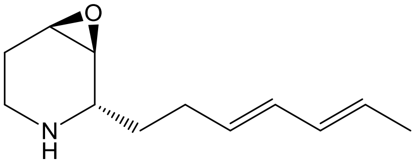

argimycin PXI

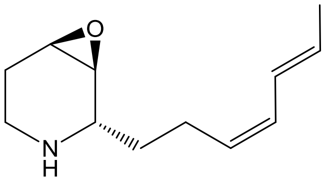

argimycin PX

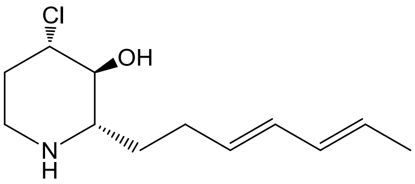

argimycin PXII

Figure S11: ^{1}H-NMR spectrum of argimycin PVII (CD_3OD, 500 MHz). A: overall chart. B: expansion of the olefinic region. C: expansion of the aliphatic region.

A

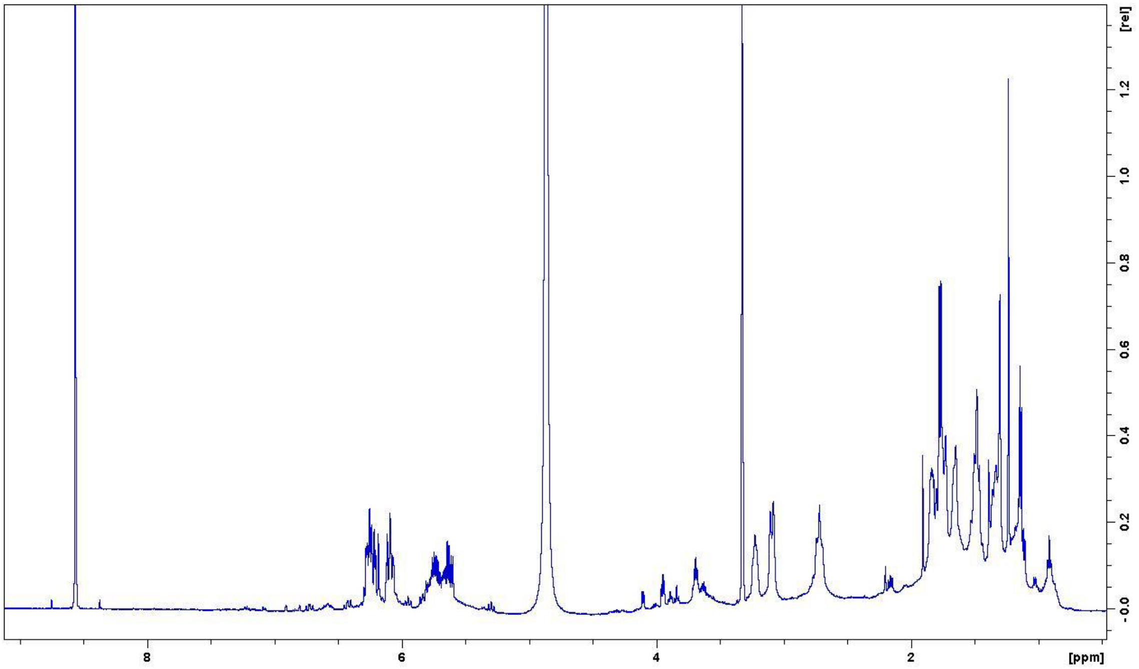

B

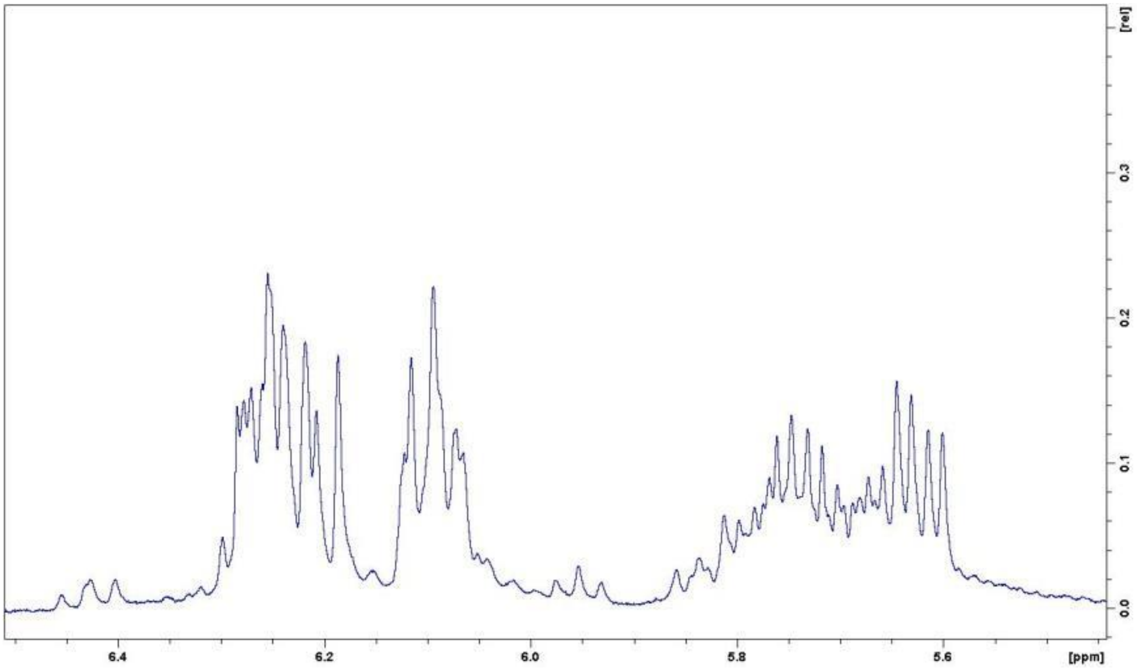

C

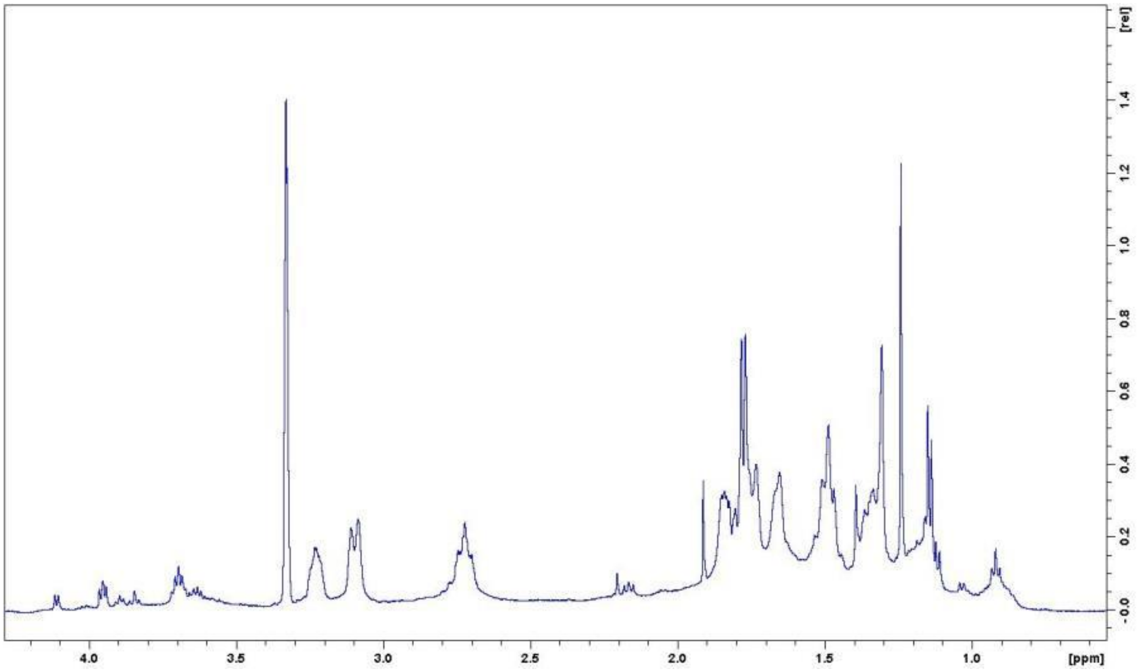

Figure S12: HSQC spectrum of argimycin PVII

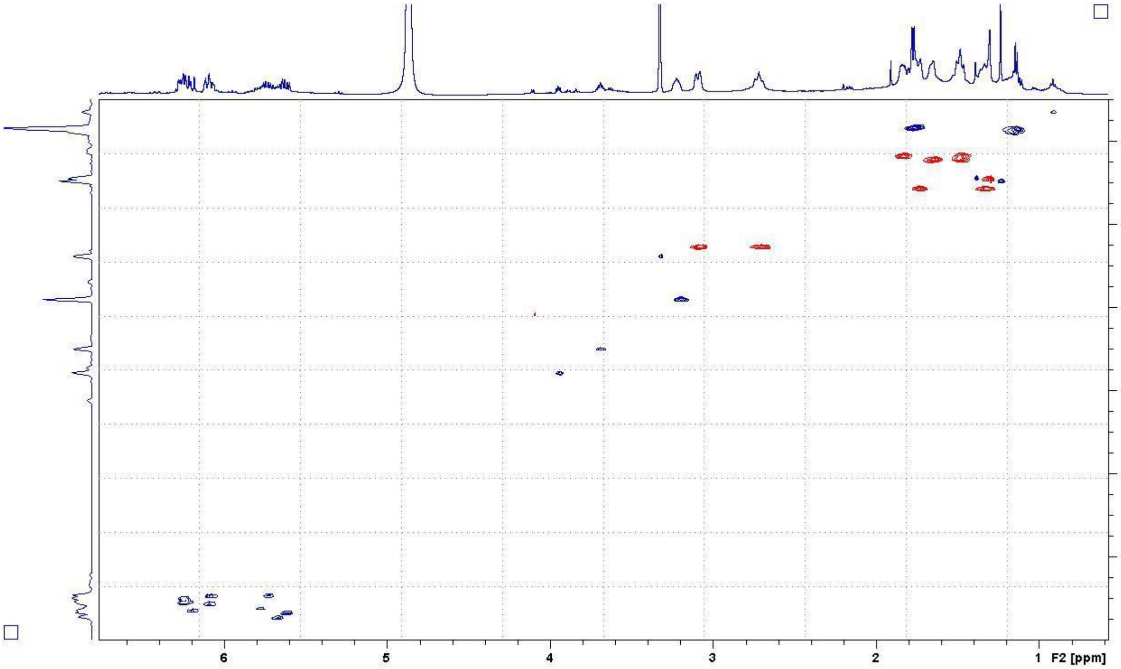

Figure S13: ^{1}H-NMR spectrum of argimycin PVIII and argimycin PXII (CD_3OD, 500 MHz). A: overall chart. B: expansion of the olefinic region. C: expansion of the aliphatic region.

A

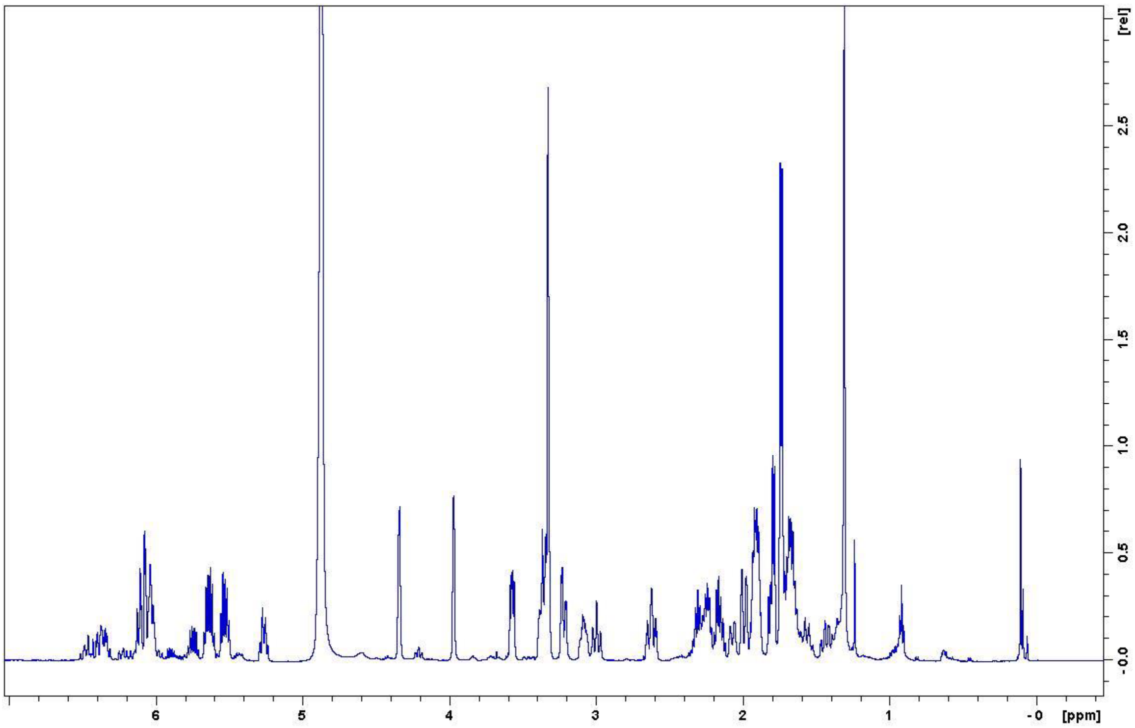

B

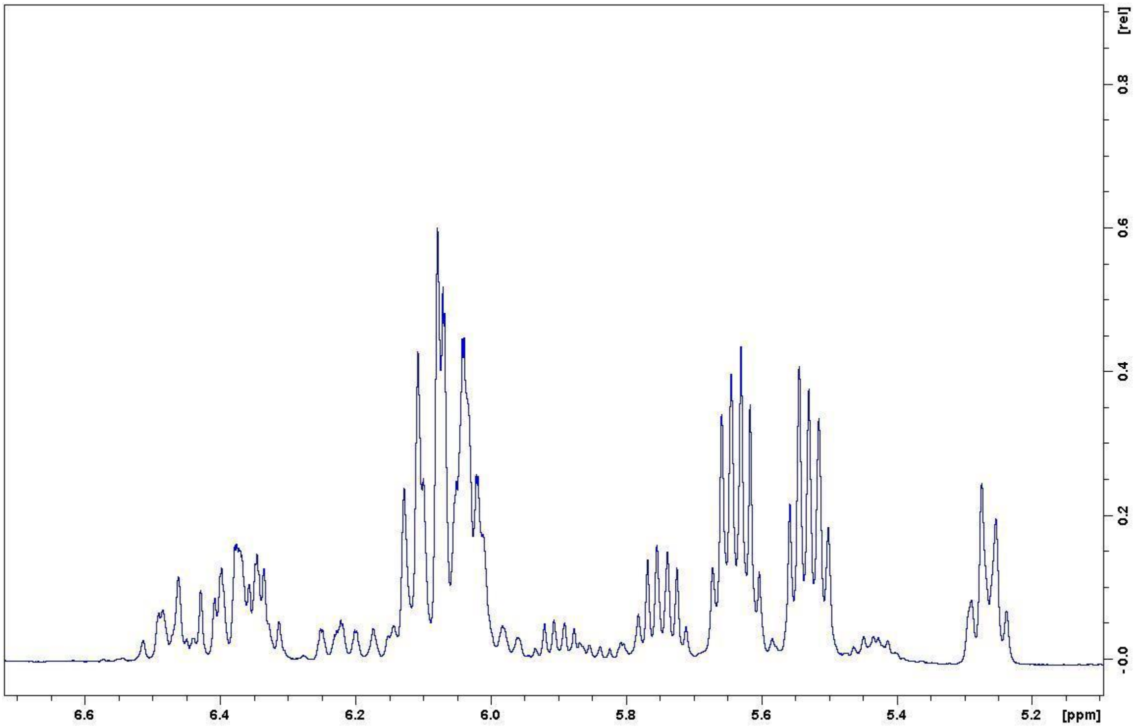

C

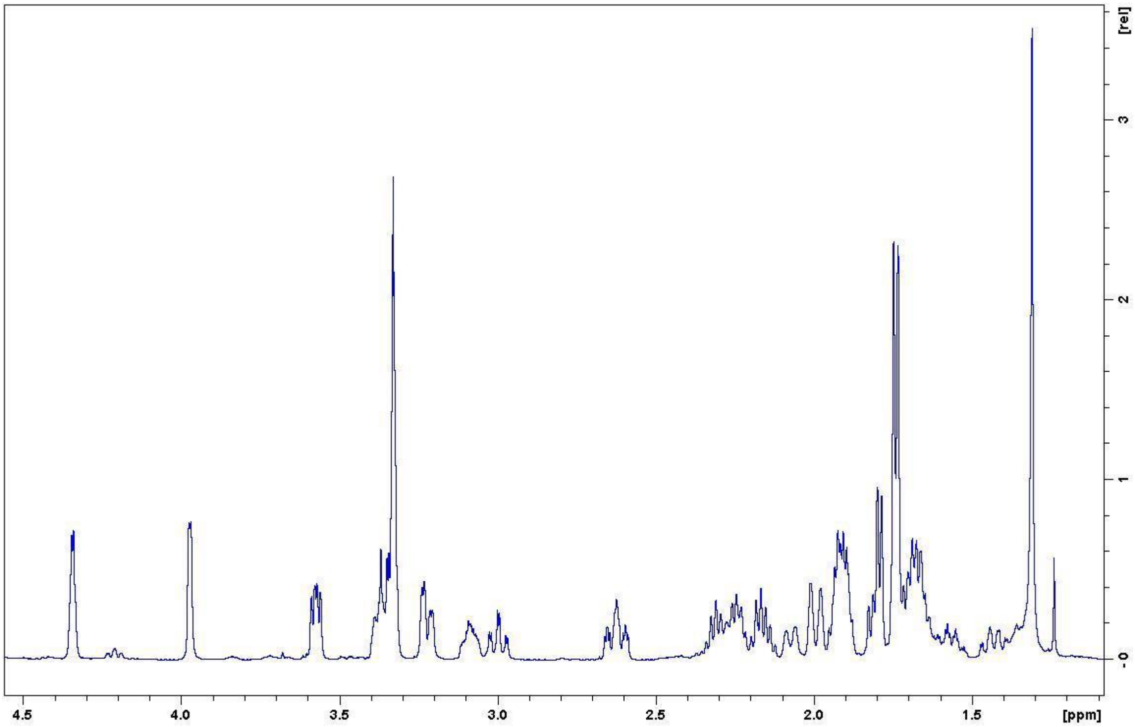

Figure S14: HSQC spectrum of argimycin PVIII and argimycin PXII

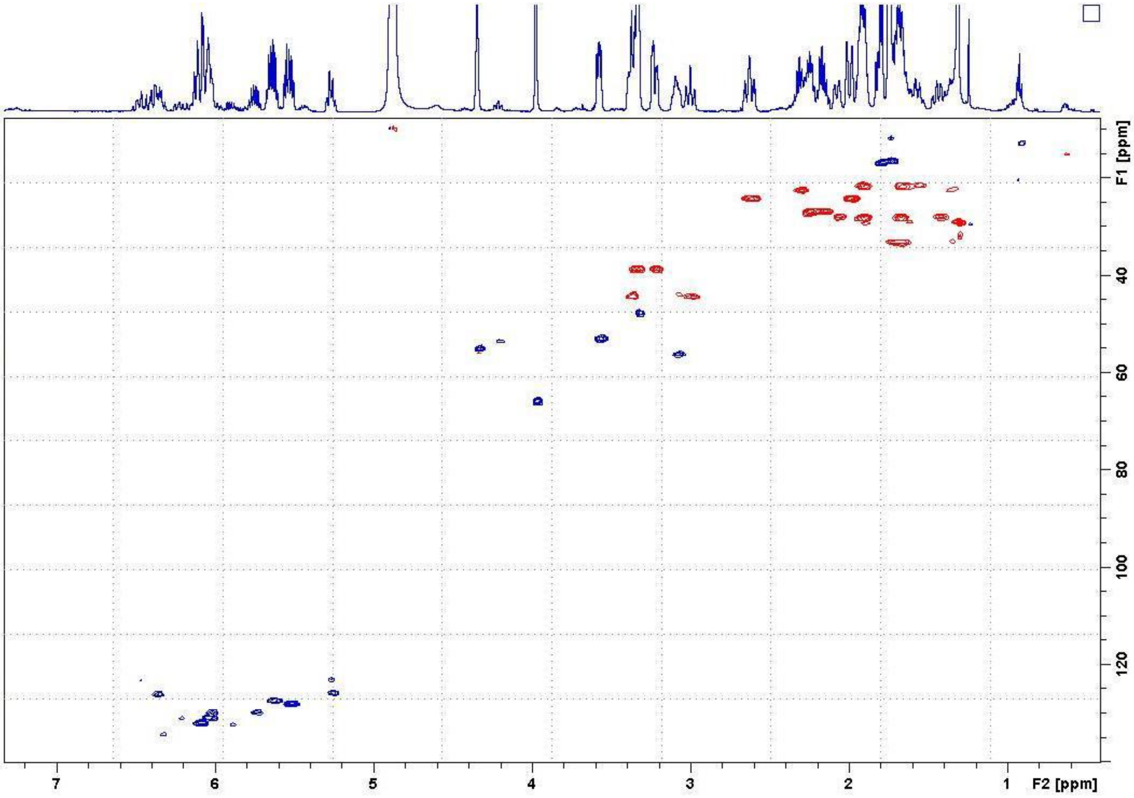

Figure S15: Generation of mutant MARPO. (A) Scheme representing the replacement event for generation of mutant MARPO. WT, wild type strain; *aac(3)IV*, apramycin resistance gene; blue rectangles in pHZMutorf5 represent DNA regions flanking the apramycin resistance cassette, which include those genes highlighted within dotted rectangles in the chromosomal DNA of the WT strain (B) PCR analysis of MARPO mutant. PCR products from the WT strain and from MARPO mutant (lanes 1, 2), using oligonucleotides Mutorf5C_A/Mutorf5C_B. λ, PstI-digested Lambda DNA

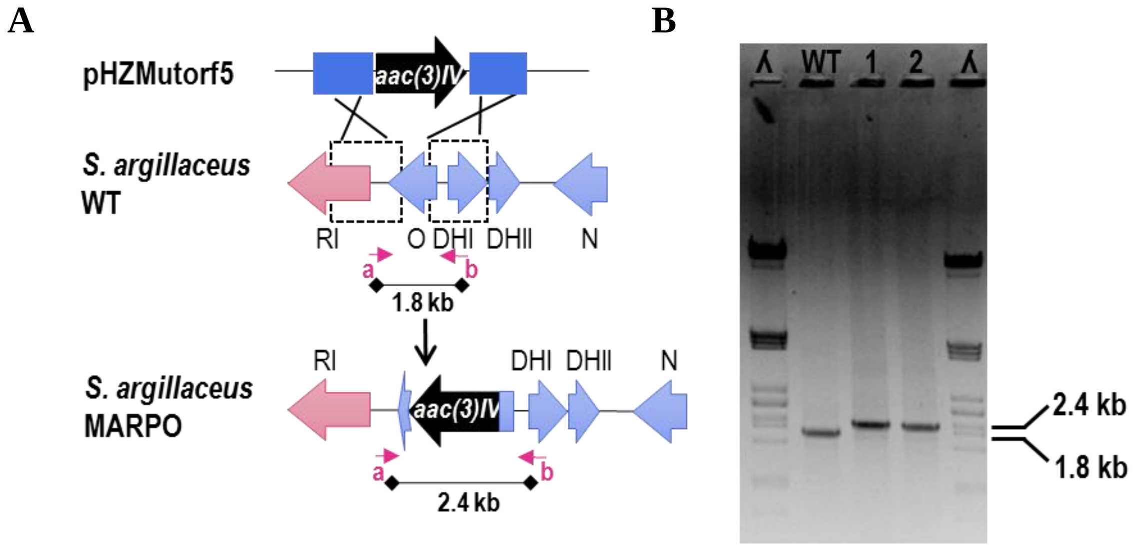

Figure S16: ^{1}H-NMR spectrum of argimycin PXIV (CD_3OD, 500 MHz).

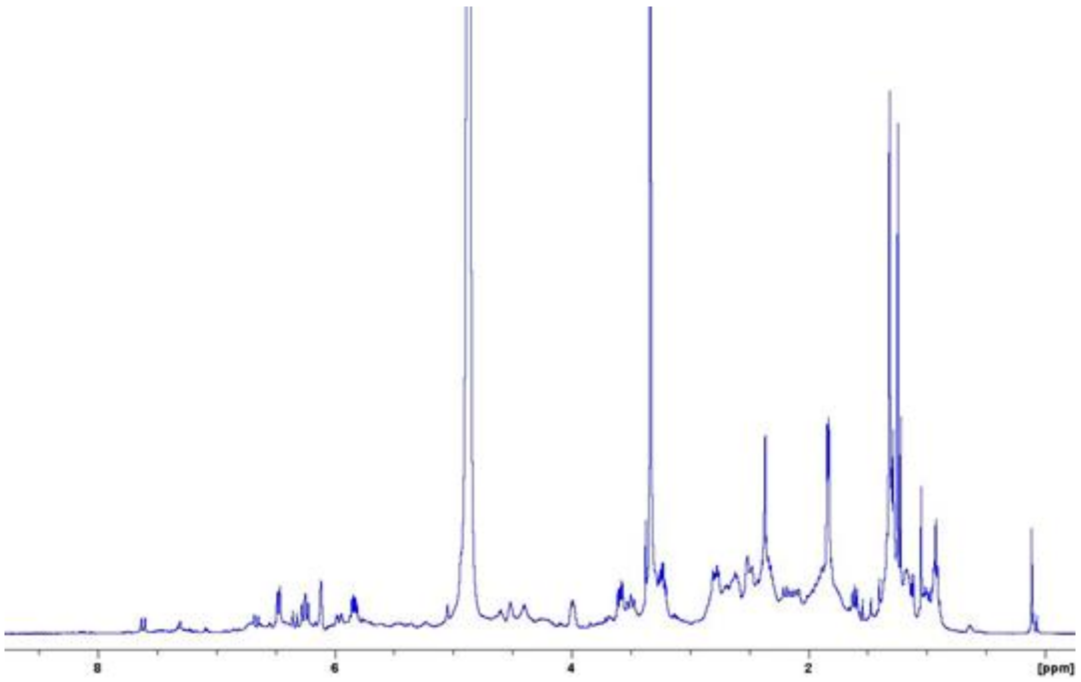

Figure S17: HSQC spectrum of argimycin PXIV

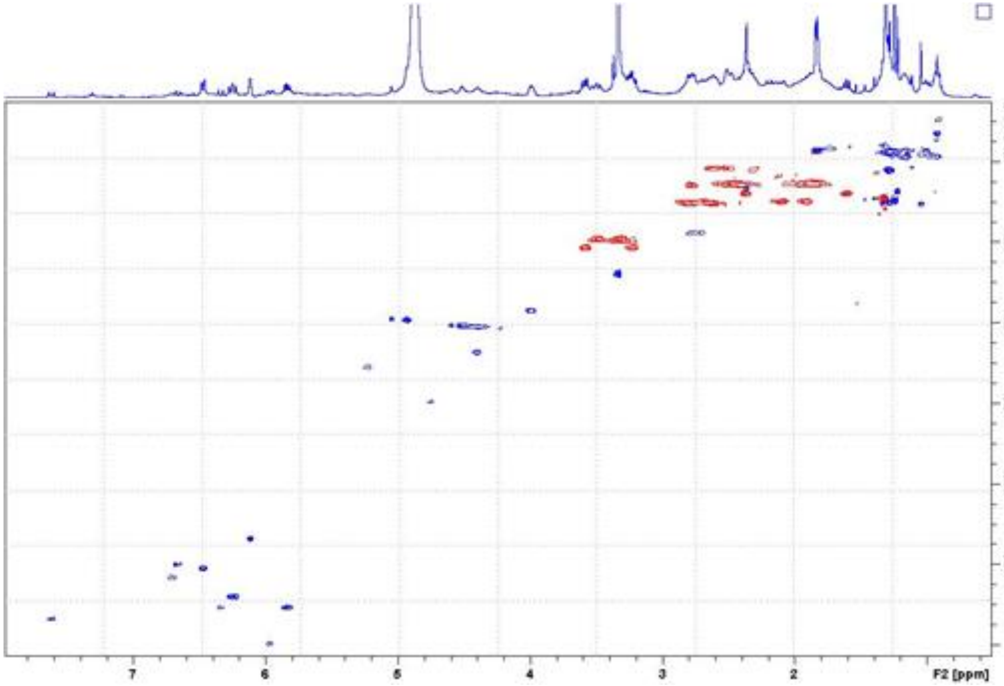

Figure S18: Left: Key HMBC correlations and a key allylic correlation observed in the COSY spectrum (dashed double arrow) of argimycin PXIV; Right: Key NOESY correlations of argimycin PXIV

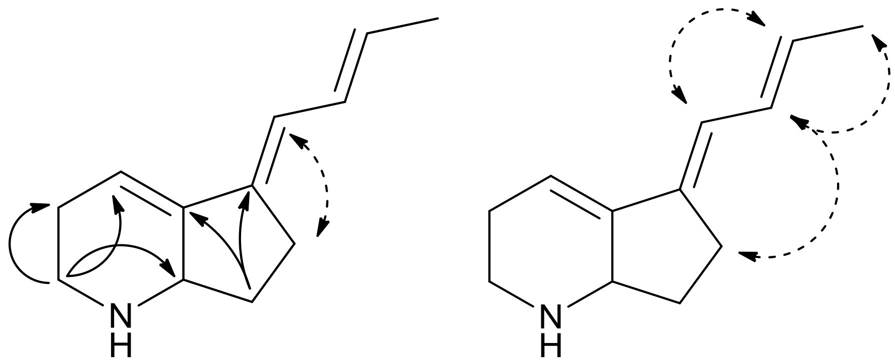

Figure S19: Generation of mutant MARPK. (A) Scheme representing the replacement event for generation of mutant MARPK. WT, wild type strain; *aac(3)IV*, apramycin resistance gene; blue rectangles in pHZMutorf9 represent DNA regions flanking the apramycin resistance cassette, which include those genes highlighted within dotted rectangles in the chromosomal DNA of the WT strain (B) PCR analysis of MARPK mutant. PCR products from the WT strain and from MARPK mutant (lanes 1-3), using oligonucleotides MutAT1_B and Mutorf9a_1_B. λ, PstI-digested Lambda DNA.

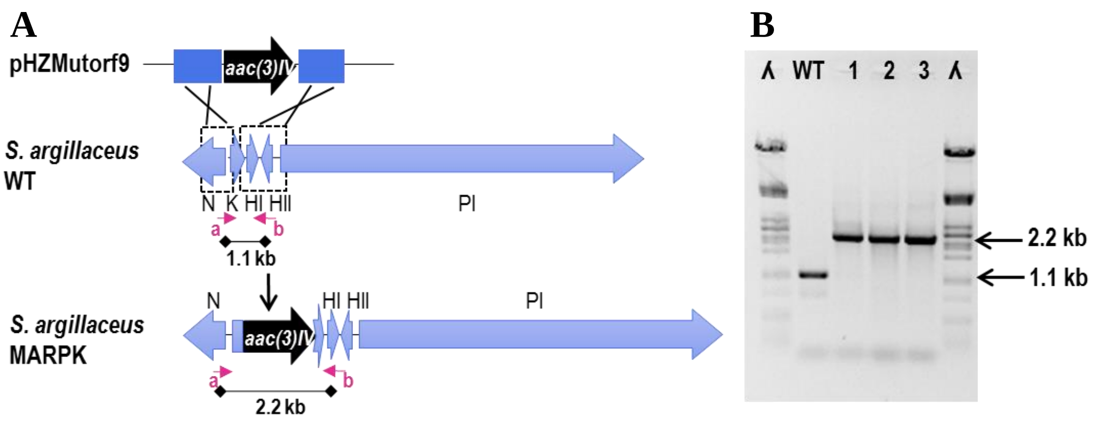

Figure S20: Generation of mutant MARPX. (A) Scheme representing the replacement event for generation of mutant MARPX. WT, wild type strain; *aac(3)IV*, apramycin resistance gene; blue rectangles in pHZMutorf14 represent DNA regions flanking the apramycin resistance cassette, which include those genes highlighted within dotted rectangles in the chromosomal DNA of the WT strain (B) PCR analysis of MARPX mutant. PCR products from the WT strain and from MARPX mutant (lanes 1, 2), using oligonucleotides MutTetR_2_A/MutTetR_2_B. λ, PstI-digested Lambda DNA.

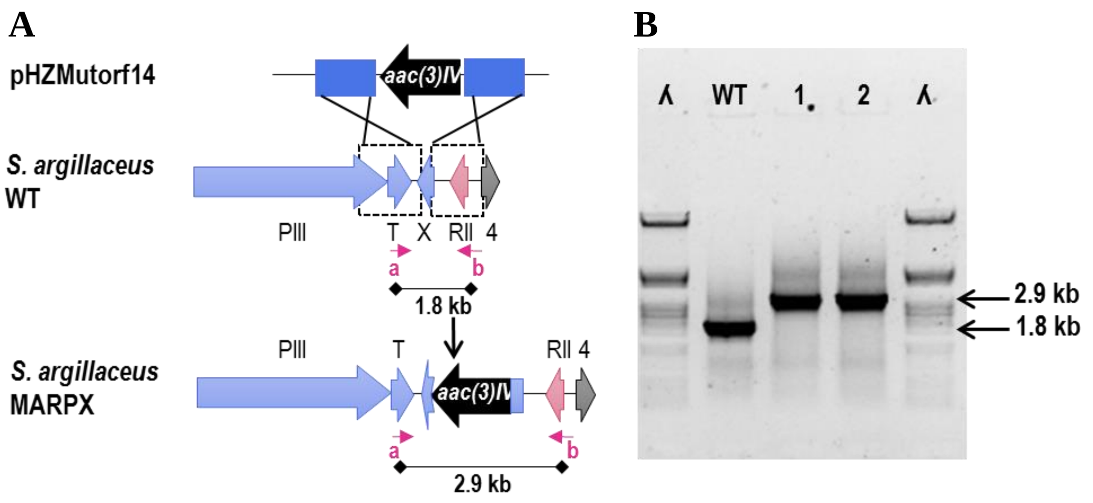

Figure S21: Proposed biosynthesis of argimycins P: initial steps

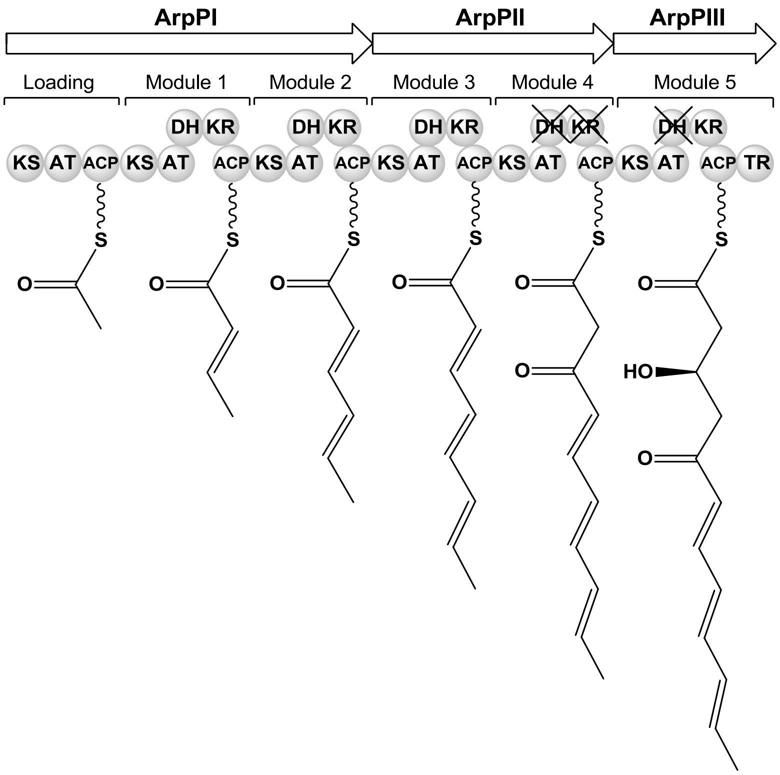

Figure S22: Argimycins P production by *S. argillaceus* WT and regulatory mutant strains (A) and by *S. argillaceus* WT overexpressing regulatory genes (B). Bars correspond to argimycins P production in arbitrary units by *S. argillaceus* WT and *S. argillaceus* WT expressing pSETE (white bars); *S. argillaceus* MARPRI and *S. argillaceus* WT expressing *arpRI* (grey bars); and *S. argillaceus* MARPRII and *S. argillaceus* WT expressing *arpRII* (black bars). Values represent the mean±SD of three independent experiments. Quantified argimycins P are mentioned in the figure as follows: **I/II**, argimycins PI/PII; **N**, nigrifactin; **PV**, argimycin PV; **PVI**, argimycin PVI.

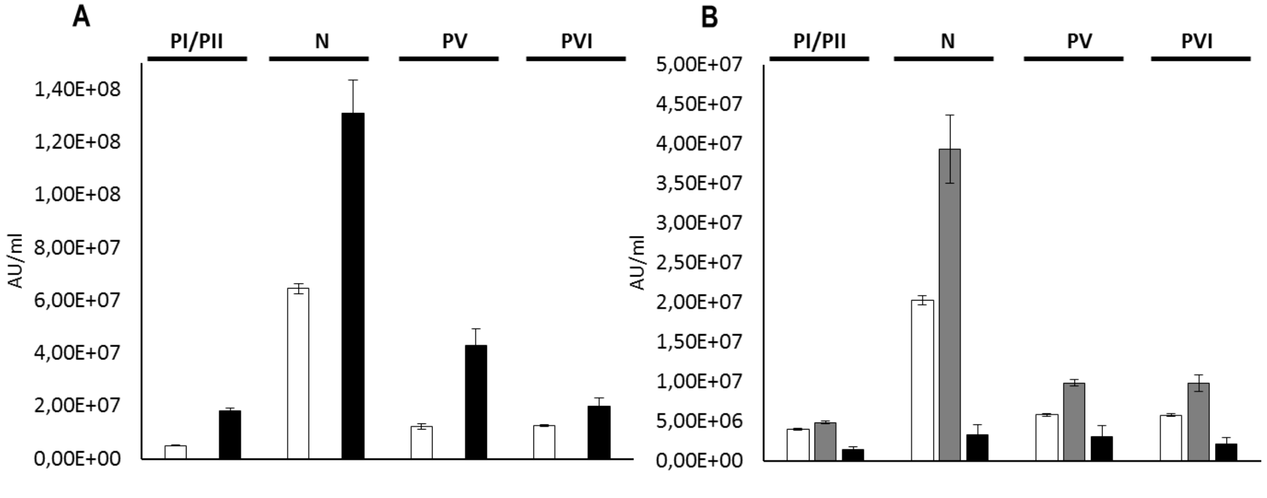

Figure S23: Comparison of *arp* gene cluster from *S. argillaceus*, and the *stz* and PKS clusters from *Streptomyces* sp. MSC090213JE08 and *Streptomyces* sp. NRRL S-1022 respectively.

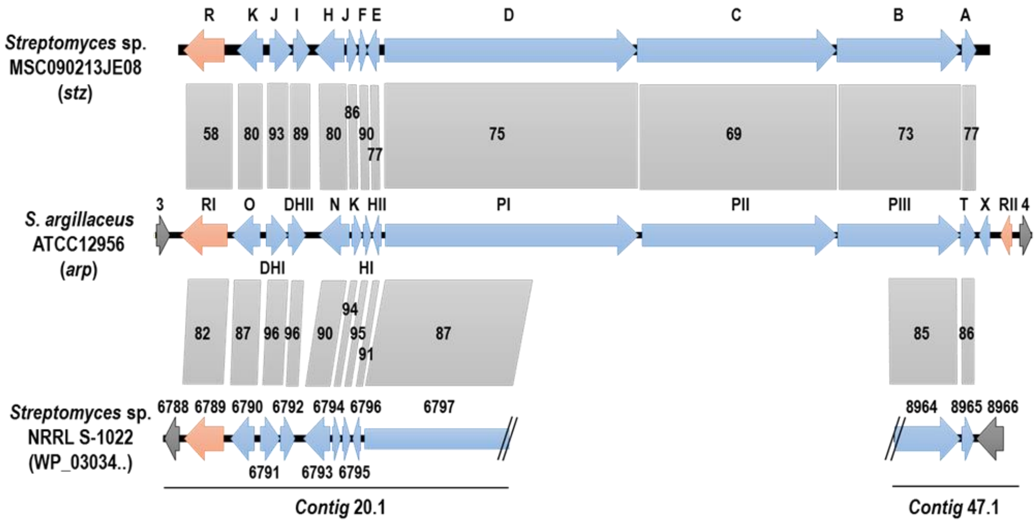

Figure S24: Original gels for Figure 7A: Transcription analysis by RT-PCR of *arp* genes in the wild type (WT) strain (gels A-C), MARPRII (gels D-F) and MARPRI (gels G-I). cDNA synthesis was performed using 50 ng of total RNA (gels A, B, D, E, G and H) and 100 ng (gels C, F and I). Gel J shows RT-PCR products of *arpDHI* using 50 ng of RNA, in the WT, MARPRII and MARPRI; RT, RT-PCR; C, negative control.

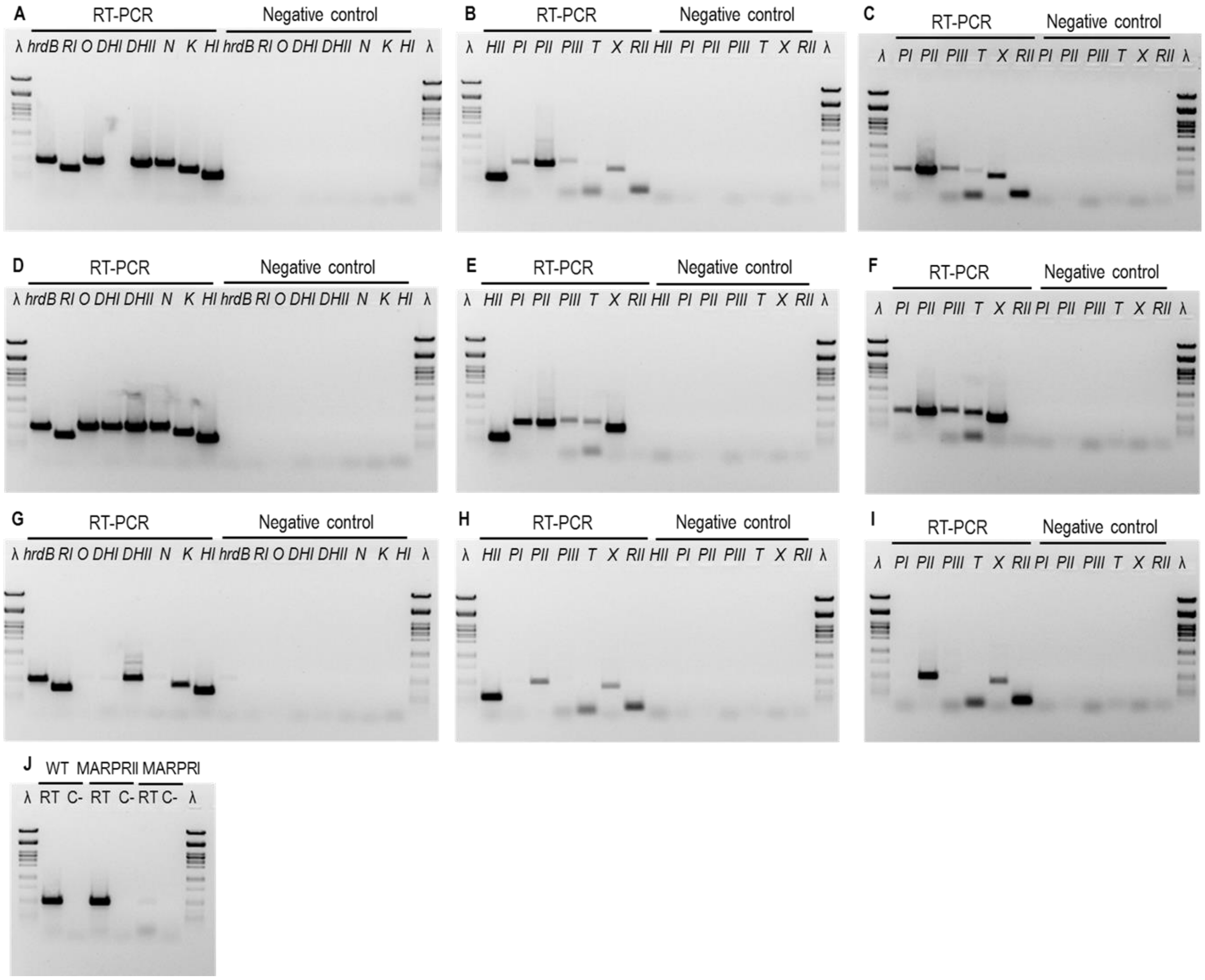

Supplement: Supplementary file 1 [file Presentation_1.PDF]
